# Prognostic pan-cancer and single-cancer models: A large-scale analysis using a real-world clinico-genomic database

**DOI:** 10.1371/journal.pone.0341355

**Published:** 2026-02-13

**Authors:** Sarah F. McGough, Svetlana Lyalina, Devin Incerti, Yunru Huang, Stefka Tyanova, Kieran Mace, Chris Harbron, Ryan Copping, Balasubramanian Narasimhan, Robert Tibshirani

**Affiliations:** 1 Computational Sciences, Genentech Research and Early Development, Genentech, Inc., South San Francisco, California, United States of America; 2 Safety Science, Product Development, Genentech, Inc., South San Francisco, California, United States of America; 3 Data Sciences, Product Development, Genentech, Inc., South San Francisco, California, United States of America; 4 Data Sciences, Product Development, F. Hoffmann-La Roche, Ltd., Welwyn Garden City, United Kingdom; 5 Department of Statistics, Stanford University, Stanford, California, United States of America; 6 Department of Biomedical Data Sciences, Stanford University, Stanford, California, United States of America; The University of Queensland Faculty of Medicine, AUSTRALIA

## Abstract

Prognostic models in oncology have a profound impact on personalized cancer care and patient profiling, but tend to be heterogeneously developed and implemented in narrow patient cohorts. Here, we develop and benchmark multiple machine learning models to predict survival in pan-cancer and 16 single-cancer settings using a de-identified clinico-genomic database of 28,079 US patients with cancer. We identify key predictors of cancer prognosis, including 15 shared across seven or more cancer types, revealing strong consistency in cancer prognostic factors. We demonstrate that pan-cancer models generally outperform or match single-cancer models in predicting survival and risk stratifying patients, especially in smaller cancer cohorts, suggesting a unique transfer learning advantage of pan-cancer models. This work demonstrates the potential of pan-cancer approaches in enhancing the accuracy and applicability of prognostic models in oncology, paving the way for more personalized and effective cancer care strategies.

## Introduction

Prognostic models — models which predict a future health state, like survival — have a direct and important impact in precision oncology. In clinical practice, prognosis informs personalized treatment and care management by helping identify the future course of illness, appropriate course of therapy (i.e., ranging from aggressive treatment to surveillance), and resource allocation [1]. In clinical studies, stratifying patients into prognostic risk categories can aid in patient recruitment and trial enrichment for high-risk patients [2,3]. And critically, prognostic models have a profound impact on patient care, with these strategies enhancing quality of life and care by guiding clinicians towards the best treatment options tailored to each patient’s unique health profile. Typically, prognostic models are developed using a few disease-specific prognostic factors collected in routine clinical practice and used to predict patient survival or risk of death [4–6].

The recent availability of large volumes of longitudinal, highly curated, and often linked patient-level health data from digital sources such as electronic health records (EHR) and genomic sequencing is contributing to advances in precision medicine by routinely collecting and storing millions of data points that offer a much more comprehensive patient profile. Machine learning models can learn from these high-dimensional datasets more effectively, bringing an opportunity for researchers to develop prognostic models that better leverage the myriad of prognostic factors from the patient’s health profile – not only illuminating key drivers in patient prognosis, but also driving improved and more personalized patient care.

Separately, an emerging paradigm in oncology is that of *pan-cancer* (cancer agnostic) research and treatment, in which cancer is characterized by genetic and molecular features rather than by its site of origin in the body. Indeed, multiple therapies have been approved in the last 5 years to treat a collection of cancer types on the basis of shared genetic mutations or predictive biomarkers that have been discovered to benefit from targeted treatment, such as tumor mutational burden (TMB) [7] and fusions on the *NTRK* gene [8]. Pan-cancer prognostic factors –- particularly genomic or molecular in origin –- are another area of research that have shown early promise but warrant deeper exploration [9]. Further, whether pan-cancer settings provide a unique learning opportunity for prognostic models, over those typically developed in single cancer settings, is unknown [10].

Although real-world prognostic models have been developed in the literature, a majority have been constructed using either clinical [9,11] or genomic [12] data alone, or within specific disease settings [13]. To advance our understanding of pan-cancer prognosis, it is essential to broaden the scope. Here we access a large, heterogeneous, and multi-cancer clinico-genomic database that offers a powerful tool for understanding both cancer genomics and clinical factors that impact survival under a pan-cancer paradigm. To our knowledge, the present study is the first large-scale analysis combining clinical and genomic data to evaluate and compare predictions in pan-cancer and dozens of single-cancer settings.

Our contributions are as follows: we systematically build and benchmark multiple pan-cancer and single-cancer machine learning prognostic models ranging in complexity using a large real-world clinico-genomic database; we identify key pan-cancer and single-cancer factors both shared and unique to each patient setting; and finally, on the basis of these factors, we risk stratify patients into prognostic subgroups. We compare the performance of pan- and single-cancer models to assess where pan-cancer models can provide advantage, and discuss implications for clinical and research settings.

## Results

### Pan- and single-cancer systematic modeling framework

**Fig 1:**
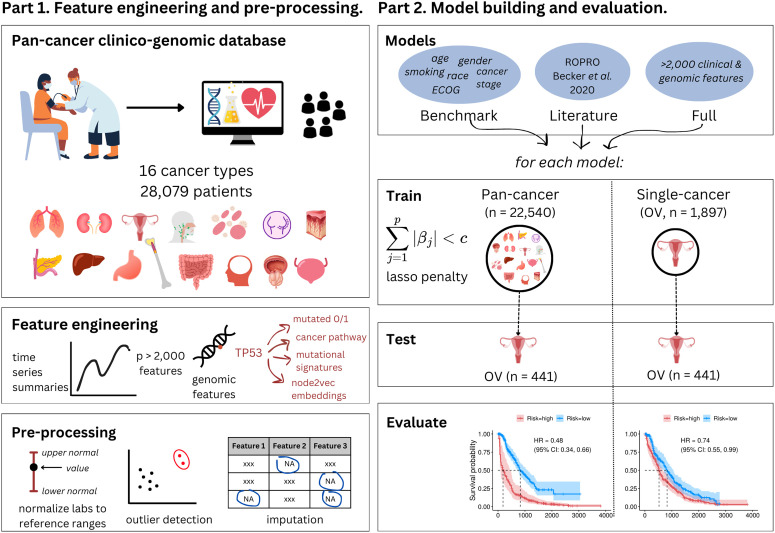
Pan- and single-cancer modeling framework. A US nationwide clinico-genomic database containing 28,079 patients and 16 cancer types was used to engineer over 2,000 features representing different modalities including demographics, treatment, laboratory tests and vital signs (represented as time series summaries), and genomics (represented as binary mutation status, affected cancer pathway, mutational signatures, and node2vec embeddings). Data were uniformly pre-processed including steps like outlier detection and processing and imputation. All steps are described in detail in *Materials & Methods* and are performed separately in train and test data where appropriate. Following this, multiple models were constructed with different feature sets: “benchmark” containing simple clinical features; “ROPRO” containing clinical features validated in the literature; “full” containing all 2,135 one-hot encoded clinico-genomic features. These models were trained both in pan-cancer and single-cancer cohorts, and evaluated in a single-cancer, out-of-sample test set for their ability to predict survival and risk stratify patients into high- and low-risk groups.

We endeavored to create a systematic, reproducible framework for the building and benchmarking of multiple pan- and single-cancer prognostic models, outlined in Fig 1. This framework represents an end-to-end, data-driven process governing feature engineering, model building, model prediction, and model evaluation to enable comparisons between models and between cancer settings.

We obtained retrospective data on 28,079 patients from 16 different cancer cohorts with a recorded first line of therapy (1L) between January 1, 2011 and June 30, 2020 in a US clinico-genomic database (CGDB) linking longitudinal, patient-level electronic health records (EHRs) with patient-level tumor genomic profiling of >300 cancer-related genes [14,15].

For each patient, we derived over 2,000 features representing 5 data modalities: clinical/demographic, laboratory/vital signs, treatment, cancer-specific (these 4 modalities collectively referred to as “clinical”), and genomic (Table S1 in S1 File). For each of the hundreds of individual lab tests in the database, we computed multiple time series summaries up until the point of prediction (1L initiation date). Genomic data, which contributed a majority of features, were used to characterize: (i) the alteration status (mutated or wild type) of >300 cancer-related genes for three variant types (short variant, copy number, and rearrangement), (ii) cancer biology pathways affected by these alterations, (iii) mutational signatures defined by the Catalogue Of Somatic Mutations In Cancer (COSMIC), and (iv) underlying protein interaction networks of affected genes (“node2vec”). In total, 2,059 features were derived (increasing to 2,135 model input features after one-hot encoding).

The pan-cancer cohort was highly heterogeneous with respect to cancer type and key clinico-genomic factors (Table 1). A majority of patients had solid tumor cancer diagnoses such as non-small cell lung cancer (NSCLC, n = 7,157, 25.4%), colorectal cancer (CRC, n = 5,059, 18.0%), and breast cancer (n = 4,801, 17.1%). A vast majority of all patients (n = 25,138, 89.5%) were diagnosed with advanced or metastatic disease by the time of 1L initiation. Patient sample sizes for hematological (blood, bone marrow, and lymph node) diagnoses were considerably smaller, notably for diffuse large B-cell lymphoma (DLBCL, n = 163, 0.6%) and chronic lymphocytic leukemia (CLL, n = 109, 0.4%). Patient age ranged from 18 to 85 (median: 64 years; interquartile range, (IQR): 56, 72 years) and the median year of frontline therapy was 2017 (IQR: 2015, 2018). Approximately 42% of the pan-cancer cohort were never-smokers, though this ranged from 4% to 62% across individual cancer cohorts. Aligning with well-studied cancer biology, the TP53 gene short variant (SV), which encodes a tumor suppressor protein, was the most frequent alteration in the pan-cancer cohort with 62.5% of all patients having the alteration.

**Table 1:** Cohort characteristics.

	Pan cancer (N=28,079)	Breast (N=4,801)	CLL (N=109)	Colorectal (N=5,059)	DLBCL (N=163)	Gastric (N=1,712)	Head and Neck (N=385)	Hepatocellular Carcinoma (N=183)	Melanoma (N=717)	Multiple Myeloma (N=325)	Non-Small Cell (N=7,157)	Ovarian (N=2,338)	Pancreatic (N=1,918)	Prostate (N=1,235)	Renal (N=648)	Small Cell (N=446)	Urothelial (N=883)
**Age**																	
**Mean (SD)**	63.2 (11.8)	58.1 (12.2)	65.1 (9.79)	59.9 (12.2)	61.7 (14.9)	63.4 (11.6)	63.7 (9.68)	62.4 (12.5)	63.2 (13.4)	61.5 (10.1)	67.2 (10.4)	62.9 (11.1)	64.9 (9.75)	68.9 (8.31)	61.2 (11.6)	64.9 (9.51)	68.3 (9.91)
**Median (Q1, Q3)**	64.0 (56.0, 72.0)	59.0 (49.0, 67.0)	66.0 (58.0, 72.0)	60.0 (52.0, 69.0)	64.0 (53.5, 72.0)	65.0 (56.0, 71.0)	64.0 (57.0, 70.0)	63.0 (57.0, 71.0)	65.0 (55.0, 73.0)	62.0 (55.0, 69.0)	68.0 (60.0, 75.0)	64.0 (56.0, 71.0)	66.0 (58.3, 72.0)	69.0 (63.0, 75.0)	62.0 (55.0, 70.0)	65.0 (58.0, 71.0)	69.0 (62.0, 76.0)
**Index Year (Year at First Line of Therapy)**																	
**Mean (SD)**	2016 (2.86)	2016 (2.47)	2016 (2.26)	2015 (3.38)	2015 (3.38)	2017 (1.95)	2017 (1.92)	2016 (2.30)	2017 (1.94)	2014 (3.16)	2017 (2.09)	2016 (2.52)	2017 (1.86)	2017 (2.04)	2016 (2.51)	2016 (2.02)	2017 (2.04)
**Median (Q1, Q3)**	2017 (2015, 2018)	2016 (2014, 2018)	2016 (2014, 2018)	2017 (2015, 2018)	2016 (2014, 2017)	2017 (2016, 2018)	2017 (2016, 2018)	2016 (2015, 2018)	2017 (2016, 2019)	2015 (2012, 2017)	2017 (2016, 2018)	2016 (2014, 2018)	2017 (2016, 2019)	2017 (2015, 2018)	2016 (2014, 2018)	2017 (2015, 2018)	2017 (2015, 2019)
**Time from diagnosis to frontline therapy (days)**																	
**Mean (SD)**	740 (1340)	1690 (2000)	1910 (2100)	551 (836)	114 (718)	214 (417)	660 (770)	357 (598)	1220 (1720)	865 (1560)	318 (644)	658 (1340)	262 (465)	1890 (1940)	904 (1480)	81.4 (274)	634 (1050)
**Median (Q1, Q3)**	157 (33.0, 836)	999 (257, 2390)	1290 (311, 2640)	161 (38.0, 778)	18.0 (8.00, 33.0)	42.0 (25.0, 233)	447 (69.0, 897)	140 (41.0, 421)	611 (86.0, 1530)	118 (33.0, 872)	54.0 (29.0, 327)	49.0 (25.0, 789)	65.0 (20.0, 367)	1140 (447, 2830)	265 (56.0, 1060)	20.0 (12.0, 32.0)	261 (61.5, 674)
**Gender**																	
**F**	15548 (55.4%)	4750 (98.9%)	34 (31.2%)	2307 (45.6%)	62 (38.0%)	423 (24.7%)	70 (18.2%)	46 (25.1%)	237 (33.1%)	135 (41.5%)	3601 (50.3%)	2338 (100%)	878 (45.8%)	0 (0%)	181 (27.9%)	220 (49.3%)	266 (30.1%)
**M**	12531 (44.6%)	51 (1.1%)	75 (68.8%)	2752 (54.4%)	101 (62.0%)	1289 (75.3%)	315 (81.8%)	137 (74.9%)	480 (66.9%)	190 (58.5%)	3556 (49.7%)	0 (0%)	1040 (54.2%)	1235 (100%)	467 (72.1%)	226 (50.7%)	617 (69.9%)
**Race**																	
**White**	20532 (73.1%)	3407 (71.0%)	67 (61.5%)	3579 (70.7%)	119 (73.0%)	1235 (72.1%)	302 (78.4%)	107 (58.5%)	613 (85.5%)	244 (75.1%)	5215 (72.9%)	1765 (75.5%)	1447 (75.4%)	896 (72.6%)	498 (76.9%)	358 (80.3%)	680 (77.0%)
**Nonwhite**	7547 (26.9%)	1394 (29.0%)	42 (38.5%)	1480 (29.3%)	44 (27.0%)	477 (27.9%)	83 (21.6%)	76 (41.5%)	104 (14.5%)	81 (24.9%)	1942 (27.1%)	573 (24.5%)	471 (24.6%)	339 (27.4%)	150 (23.1%)	88 (19.7%)	203 (23.0%)
**BMI**																	
**Normal and underweight**	11286 (40.2%)	1722 (35.9%)	34 (31.2%)	1793 (35.4%)	56 (34.4%)	817 (47.7%)	198 (51.4%)	60 (32.8%)	195 (27.2%)	114 (35.1%)	3288 (45.9%)	1090 (46.6%)	956 (49.8%)	268 (21.7%)	202 (31.2%)	163 (36.5%)	330 (37.4%)
**Overweight**	9330 (33.2%)	1514 (31.5%)	44 (40.4%)	1679 (33.2%)	62 (38.0%)	547 (32.0%)	127 (33.0%)	61 (33.3%)	293 (40.9%)	115 (35.4%)	2353 (32.9%)	661 (28.3%)	636 (33.2%)	502 (40.6%)	240 (37.0%)	166 (37.2%)	330 (37.4%)
**Obese**	7463 (26.6%)	1565 (32.6%)	31 (28.4%)	1587 (31.4%)	45 (27.6%)	348 (20.3%)	60 (15.6%)	62 (33.9%)	229 (31.9%)	96 (29.5%)	1516 (21.2%)	587 (25.1%)	326 (17.0%)	465 (37.7%)	206 (31.8%)	117 (26.2%)	223 (25.3%)
**Smoking status**																	
**History of smoking**	16313 (58.1%)	1919 (40.0%)	51 (46.8%)	2449 (48.4%)	62 (38.0%)	1127 (65.8%)	274 (71.2%)	125 (68.3%)	358 (49.9%)	126 (38.8%)	5842 (81.6%)	969 (41.4%)	991 (51.7%)	652 (52.8%)	328 (50.6%)	429 (96.2%)	611 (69.2%)
**No history of smoking**	11766 (41.9%)	2882 (60.0%)	58 (53.2%)	2610 (51.6%)	101 (62.0%)	585 (34.2%)	111 (28.8%)	58 (31.7%)	359 (50.1%)	199 (61.2%)	1315 (18.4%)	1369 (58.6%)	927 (48.3%)	583 (47.2%)	320 (49.4%)	17 (3.8%)	272 (30.8%)
**Advanced or metastatic disease**																	
**No**	2941 (10.5%)	750 (15.6%)	56 (51.4%)	102 (2.0%)	43 (26.4%)	11 (0.6%)	4 (1.0%)	0 (0%)	3 (0.4%)	107 (32.9%)	55 (0.8%)	1580 (67.6%)	81 (4.2%)	105 (8.5%)	13 (2.0%)	21 (4.7%)	10 (1.1%)
**Yes**	25138 (89.5%)	4051 (84.4%)	53 (48.6%)	4957 (98.0%)	120 (73.6%)	1701 (99.4%)	381 (99.0%)	183 (100%)	714 (99.6%)	218 (67.1%)	7102 (99.2%)	758 (32.4%)	1837 (95.8%)	1130 (91.5%)	635 (98.0%)	425 (95.3%)	873 (98.9%)
**TP53 (Short Variant) status**																	
**0**	10521 (37.5%)	2452 (51.1%)	73 (67.0%)	1216 (24.0%)	97 (59.5%)	401 (23.4%)	198 (51.4%)	118 (64.5%)	533 (74.3%)	278 (85.5%)	2571 (35.9%)	450 (19.2%)	488 (25.4%)	688 (55.7%)	552 (85.2%)	36 (8.1%)	370 (41.9%)
**1**	17558 (62.5%)	2349 (48.9%)	36 (33.0%)	3843 (76.0%)	66 (40.5%)	1311 (76.6%)	187 (48.6%)	65 (35.5%)	184 (25.7%)	47 (14.5%)	4586 (64.1%)	1888 (80.8%)	1430 (74.6%)	547 (44.3%)	96 (14.8%)	410 (91.9%)	513 (58.1%)
**Tissue TMB score**																	
**Mean (SD)**	6.98 (13.7)	4.85 (8.53)	2.31 (4.47)	6.04 (13.3)	11.9 (10.2)	6.13 (7.94)	6.10 (8.41)	4.72 (9.48)	27.9 (36.5)	4.96 (7.70)	9.47 (15.0)	3.75 (11.8)	4.04 (7.69)	4.92 (11.4)	3.44 (3.71)	8.83 (6.86)	9.17 (13.8)
**Median (Q1, Q3)**	3.75 (1.74, 7.50)	2.61 (1.25, 5.22)	1.61 (0.807, 2.42)	3.48 (2.40, 5.22)	8.88 (4.84, 15.3)	4.35 (2.50, 6.96)	3.75 (2.40, 7.50)	3.48 (1.74, 5.22)	15.6 (5.22, 34.8)	3.23 (1.25, 6.46)	6.25 (2.61, 11.3)	2.61 (1.25, 4.35)	2.50 (1.25, 3.75)	2.50 (1.25, 4.35)	2.61 (1.25, 4.35)	7.50 (4.85, 11.3)	6.25 (3.48, 11.3)
**PDL1 status**																	
**0**	25913 (92.3%)	4639 (96.6%)	99 (90.8%)	4690 (92.7%)	148 (90.8%)	1395 (81.5%)	322 (83.6%)	168 (91.8%)	644 (89.8%)	297 (91.4%)	6673 (93.2%)	2240 (95.8%)	1789 (93.3%)	1112 (90.0%)	601 (92.7%)	418 (93.7%)	678 (76.8%)
**1**	2166 (7.7%)	162 (3.4%)	10 (9.2%)	369 (7.3%)	15 (9.2%)	317 (18.5%)	63 (16.4%)	15 (8.2%)	73 (10.2%)	28 (8.6%)	484 (6.8%)	98 (4.2%)	129 (6.7%)	123 (10.0%)	47 (7.3%)	28 (6.3%)	205 (23.2%)
**Last Albumin, Abnormal**																	
**No**	24245 (86.3%)	4479 (93.3%)	104 (95.4%)	4392 (86.8%)	144 (88.3%)	1379 (80.5%)	335 (87.0%)	134 (73.2%)	649 (90.5%)	280 (86.2%)	5928 (82.8%)	1985 (84.9%)	1539 (80.2%)	1179 (95.5%)	567 (87.5%)	389 (87.2%)	762 (86.3%)
**Yes**	3834 (13.7%)	322 (6.7%)	5 (4.6%)	667 (13.2%)	19 (11.7%)	333 (19.5%)	50 (13.0%)	49 (26.8%)	68 (9.5%)	45 (13.8%)	1229 (17.2%)	353 (15.1%)	379 (19.8%)	56 (4.5%)	81 (12.5%)	57 (12.8%)	121 (13.7%)
**Last Absolute Lymphocyte Count, Abnormal**																	
**No**	20421 (72.7%)	3645 (75.9%)	37 (33.9%)	3969 (78.5%)	103 (63.2%)	1186 (69.3%)	209 (54.3%)	130 (71.0%)	552 (77.0%)	214 (65.8%)	4827 (67.4%)	1752 (74.9%)	1405 (73.3%)	893 (72.3%)	498 (76.9%)	363 (81.4%)	638 (72.3%)
**Yes**	7658 (27.3%)	1156 (24.1%)	72 (66.1%)	1090 (21.5%)	60 (36.8%)	526 (30.7%)	176 (45.7%)	53 (29.0%)	165 (23.0%)	111 (34.2%)	2330 (32.6%)	586 (25.1%)	513 (26.7%)	342 (27.7%)	150 (23.1%)	83 (18.6%)	245 (27.7%)

Given patient heterogeneity and vast differences in sample sizes of the different cancer cohorts, we sought to investigate whether models developed on the pan-cancer cohort could improve survival predictions compared to those developed in single-cancer settings, by learning from all of the available information and potential signals across cancer types. Using the full high-dimensional, clinico-genomic feature set, we developed a series of penalized Cox proportional hazards models (“Full” models) to predict survival from 1L initiation date in (i) the large pan-cancer cohort (*pan-cancer* model) and (ii) each of the 16 cancer cohorts separately (*single-cancer* models). To compare to single-cancer models, pan-cancer trained models were evaluated on each of the 16 separate cancer cohorts in addition to the pan-cancer cohort (Fig 1). We benchmarked these high-dimensional prognostic models against simpler models from clinical practice and the literature, and here we present: (1) a “benchmark” model containing cancer type, age, gender, race, smoking status, cancer stage at diagnosis, baseline Eastern Cooperative Oncology Group (ECOG) Performance Status, time from diagnosis to initiation of 1L, and time from genomic test to initiation of 1L; and (2) a model adapted from ROPRO (Real wOrld PROgnostic score) by Becker *et al*. [9], referred to as “ROPRO-like”. These models are described in Table S2 and SI Materials and Methods in S1 File.

The out-of-sample performance of each trained pan-cancer and single-cancer prognostic model was evaluated on a withheld, single-cancer test dataset containing 20% of the total patient cohort (split by stratified random sampling on cancer type), using three performance metrics to assess the discrimination and calibration of the survival predictions: concordance index (c-index), integrated Brier score (IBS), and the hazard ratio (HR) comparing survival between patients in predicted low-risk and high-risk groups based upon a median split. Bias-corrected 95% confidence intervals for the c-index and IBS were obtained via 1,000 bootstrap replicates of the train and test data [16].

### Model performance in pan- and single-cancer cohorts

**Fig 2:**
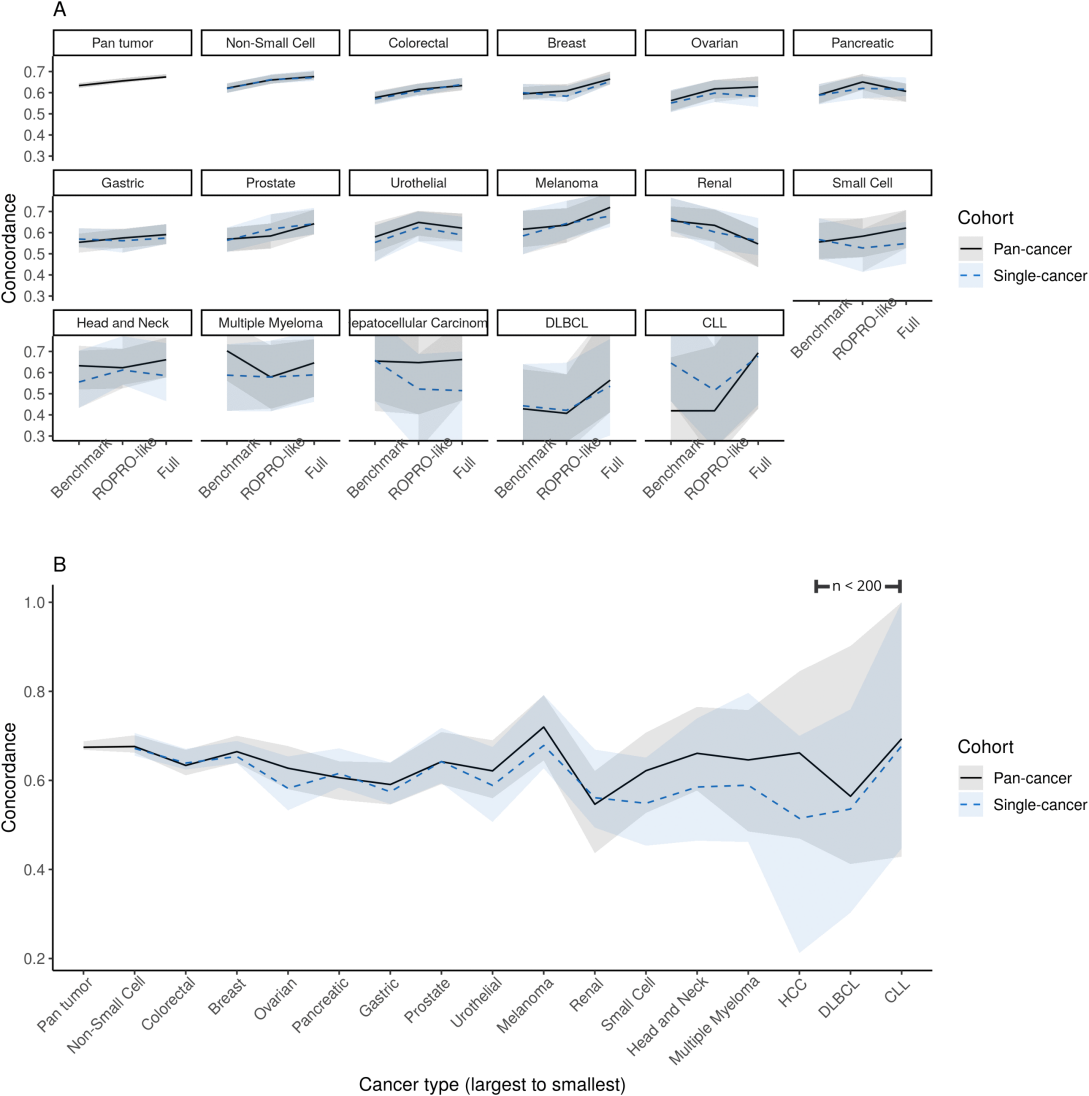
The out-of-sample concordance index (c-index), where values closer to 1 indicate higher prognostic discrimination. (A) The pan-cancer (solid black line) and single-cancer (dashed blue line) c-index for each comparator model of increasing high-dimensionality (x-axis: Benchmark, ROPRO-like, and Full models). (B) For each cancer cohort, we compare the pan-cancer (solid black line) and single-cancer (dashed blue line) c-index for the Full model constructed on the full feature set of > 2,000 features. 95% bias-corrected percentile intervals (shaded) around the estimates are shown for 1,000 bootstrap replicates. In both plots, cancer types are arranged from largest to smallest sample size.

Table 2 summarizes the out-of-sample c- index for the different comparator models evaluated on each cancer cohort. The c-index provides a measure of how well a model can discriminate prognosis between patients. For each model, the pan-cancer out-of-sample performance is presented alongside that of the equivalent single-cancer model. Fig 2 visualizes trends in pan-cancer and single-cancer c-indexes across (A) all 3 comparator models and (B) all 16 cancer cohorts for only the full high-dimensional model.

**Table 2:** C-index performance measures for single-cancer (SC) and pan-cancer (PC) models of increasing number of predictors (‘p’).

Indication	Number of events (%)	Comparator models					
		Benchmark (p = 9)		RoPro-like (p = 29)		Full Model (p = 2,059)	
		SC	PC	SC	PC	SC	PC
Pan cancer (n = 28,079)		N/A	0.63 (0.62,0.65)	N/A	0.66 (0.65,0.67)	N/A	0.673 (0.667,0.687)
Non-Small Cell Lung Cancer (n = 7,157)	4,137 (57.8%)	0.62 (0.6,0.64)	0.62 (0.6,0.65)	0.66 (0.64,0.69)	0.66 (0.64,0.68)	0.67 (0.66,0.7)	0.67 (0.66,0.7)
Colorectal (n = 5,059)	2,751 (54.4%)	0.57 (0.54,0.6)	0.58 (0.55,0.61)	0.61 (0.58,0.64)	0.62 (0.59,0.64)	0.64 (0.62,0.68)	0.63 (0.61,0.67)
Breast (n = 4,801)	2,412 (50.2%)	0.6 (0.57,0.64)	0.59 (0.56,0.63)	0.58 (0.56,0.63)	0.61 (0.58,0.64)	0.66 (0.64,0.7)	0.66 (0.64,0.7)
Ovarian (n = 2,338)	1,063 (45.5%)	0.55 (0.51,0.61)	0.56 (0.51,0.61)	0.6 (0.56,0.66)	0.62 (0.57,0.66)	0.57 (0.52,0.63)	0.62 (0.57,0.67)
Pancreatic (n = 1,918)	1,349 (70.3%)	0.59 (0.54,0.64)	0.59 (0.55,0.63)	0.62 (0.57,0.68)	0.65 (0.61,0.69)	0.62 (0.58,0.67)	0.61 (0.57,0.65)
Gastric (n = 1,712)	1,131 (66.1%)	0.57 (0.53,0.62)	0.55 (0.51,0.6)	0.56 (0.51,0.62)	0.57 (0.53,0.62)	0.57 (0.54,0.63)	0.58 (0.54,0.63)
Prostate (n = 1,235)	633 (51.3%)	0.56 (0.51,0.62)	0.57 (0.51,0.62)	0.62 (0.56,0.69)	0.58 (0.52,0.64)	0.65 (0.62,0.72)	0.63 (0.57,0.69)
Urothelial (n = 883)	527 (59.7%)	0.55 (0.46,0.63)	0.58 (0.51,0.65)	0.63 (0.56,0.7)	0.65 (0.59,0.7)	0.6 (0.52,0.67)	0.62 (0.55,0.68)
Melanoma (n = 717)	315 (43.9%)	0.58 (0.5,0.7)	0.62 (0.53,0.7)	0.64 (0.57,0.75)	0.64 (0.55,0.72)	0.66 (0.58,0.77)	0.72 (0.65,0.8)
Renal (n = 648)	332 (51.2%)	0.67 (0.61,0.77)	0.66 (0.58,0.72)	0.6 (0.52,0.71)	0.63 (0.56,0.71)	0.48 (0.36,0.57)	0.55 (0.44,0.64)
Small Cell Lung Cancer (n = 446)	321 (72.0%)	0.57 (0.48,0.67)	0.56 (0.47,0.65)	0.53 (0.41,0.62)	0.58 (0.48,0.67)	0.51 (0.39,0.59)	0.63 (0.53,0.71)
Head and Neck (n = 385)	237 (61.6%)	0.56 (0.43,0.7)	0.63 (0.52,0.73)	0.61 (0.54,0.77)	0.62 (0.53,0.71)	0.58 (0.45,0.73)	0.66 (0.57,0.76)
Multiple Myeloma (n = 325)	138 (42.5%)	0.59 (0.42,0.73)	0.7 (0.56,0.84)	0.58 (0.42,0.75)	0.58 (0.43,0.73)	0.58 (0.43,0.77)	0.63 (0.44,0.75)
Hepatocellular Carcinoma (n = 183)	112 (61.2%)	0.66 (0.47,0.86)	0.65 (0.42,0.84)	0.52 (0.23,0.69)	0.65 (0.4,0.81)	0.6 (0.32,0.82)	0.59 (0.36,0.75)
DLBCL (n = 163)	70 (42.9%)	0.44 (0.22,0.64)	0.43 (0.25,0.62)	0.42 (0.23,0.65)	0.41 (0.27,0.59)	0.54 (0.32,0.79)	0.57 (0.43,0.88)
CLL (n = 109)	24 (22.0%)	0.65 (0.47,1)	0.42 (0.16,0.67)	0.52 (0.24,0.86)	0.42 (0.19,0.72)	0.69 (0.5,1)	0.66 (0.36,1)

*Large vs. small feature set performance:* Across comparator models (benchmark, ROPRO-like, and full), there was a consistent gain in discrimination (higher c-index) for most cancer types as additional features were incorporated into the model, although this had diminishing returns for cancer cohorts with fewer patients (Fig 2A). The overall pan-cancer c-index improved slightly from the benchmark model (0.63, 95% CI: 0.62, 0.65) to the full model (0.673, 95% CI: 0.667, 0.687) with even more modest gains over the ROPRO-like model (0.66, 95% CI: 0.65, 0.67) (Table 2). Single-cancer models followed similar trends. The change in c-index was small relative to the uncertainty resulting from small sample sizes in the test set, so these findings are descriptive. However, some of the smallest cancer cohorts – with fewer than 500 patients in the training data, e.g., small-cell lung cancer, multiple myeloma, and hepatocellular carcinoma (HCC) – saw little or no improvement in the c-index as the number of features increased. Due to the wide confidence intervals and limited sample sizes, these results are descriptive and intended to illustrate general patterns rather than provide precise quantitative estimates. These findings suggest a possible performance trade-off between sample size and number of predictors.

### Pan- vs. single-cancer performance

In almost all cases, however, the pan-cancer model performed similarly to or outperformed the equivalent single-cancer models (trained on the same predictors) on the basis of the c-index, most substantially in the full (highest-dimensional) model (Fig 2B, Table 2). In particular for the full model, c-index improvements were highest among the smallest sample sizes, ranging from 2–20% higher in cancer cohorts with less than 500 patients. Meaningful gains were not observed for cancer cohorts with large sample sizes. It is worth noting that the uncertainty around these estimates is considerably high as a result of very small sample sizes, so findings are descriptive and this is observed as a general trend.

To further assess the prognostic discrimination of these models, we calculated a prognostic score for each patient using the final coefficients of each penalized Cox model, representing the predicted risk of death. We then stratified patients by cancer type into low- and high-risk categories based on the median score of the training patients in each cancer cohort.

**Fig 3:**
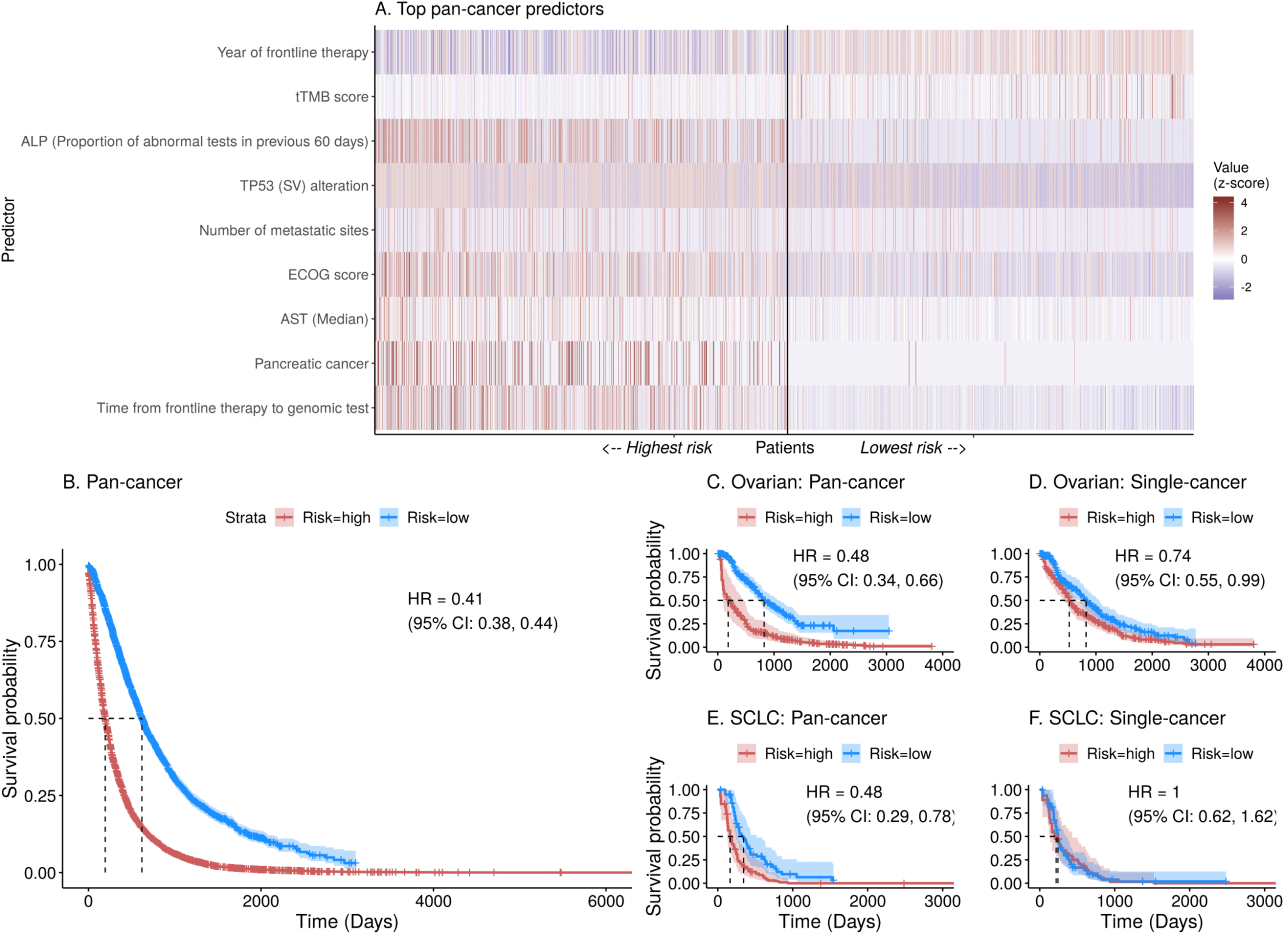
Pan-cancer and single-cancer risk stratification. (A) Heatmap showing the normalized (z-score) value of selected pan-cancer predictors in the highest (top 25%) and lowest (bottom 25%) risk patients in the out-of-sample pan-cancer cohort. (B) Out-of-sample pan-cancer risk stratification. In single-cancer cohorts, risk stratification of pan- and single-cancer models are shown for (C-D) Ovarian Cancer and (E-F) Small Cell Lung Cancer (SCLC). In (A), from left to right, patients are ordered from highest to lowest risk based on their risk scores, and their normalized value for each predictor is given in shades of red (higher) or blue (lower). A vertical line separates highest from lowest risk patients.

Aligning with trends in the c-index and sample size, the pan-cancer model outperformed single-cancer models on patient risk stratification for many cancers, most prominently for cancers with smaller sample sizes. Fig 3A visualizes key factors driving risk stratification in the pan-cancer model, such as lab tests, year of frontline therapy, tissue tumor mutational burden (tTMB), ECOG score, and TP53 (SV) alteration. In Fig 3B, the pan-cancer model yielded clear separation of the survival curve between high- and low-risk patients in the pan-cancer cohort, and training on the pan-cancer dataset generally yielded an improvement over single-cancer models (Fig 3C–3F, Figs S1–S5 in S1 File). For 12 of the 16 cancer types, hazard ratios (HRs) comparing the survival of high to low-risk patients were lower, many with narrower confidence intervals and more well-separated survival curves, using pan-cancer predictions compared to cancer-specific predictions; for example in ovarian cancer (OC), the HR was 0.48 (95% CI: 0.34, 0.66) in the pan-cancer model (Fig 3C) compared to 0.74 (95% CI: 0.55, 0.99) in the single-cancer model (Fig 3D). In small-cell lung cancer (SCLC), the pan- and single-cancer HRs were 0.48 (95% CI: 0.29, 0.78) versus 1 (95% CI: 0.62, 1.62), respectively (Fig 3E–3F).

Trends in the IBS, a measure of performance reflecting both discrimination and calibration, were less clear and presented in Fig S6 in S1 File. The integrated Brier score (IBS) summarizes how well predicted survival probabilities agree with observed outcomes over time, combining aspects of model discrimination (the ability to separate high- and low-risk patients) and calibration (the accuracy of predicted probabilities). Lower IBS values therefore indicate better overall prognostic performance. Clinically, a lower IBS reflects a model whose predicted survival curves more closely match actual patient outcomes, which may enhance its reliability for risk stratification or patient counseling. Taken together with the c-index results, the IBS results suggest that single-cancer predictions tended to be slightly better calibrated than pan-cancer predictions, in particular for the largest cancer cohorts. Similar to the c-index, the IBS improved with additional features, where improvement is marked by decreases in the score; however, the range of possible values of IBS was smaller than the uncertainty around each estimate, making interpretation of small differences difficult. For this reason, we emphasize discrimination (as measured by the c-index) as the primary indicator of model performance, with IBS results serving as a complementary measure of overall calibration quality.

### Clinico-genomic factors associated with cancer survival

**Fig 4:**
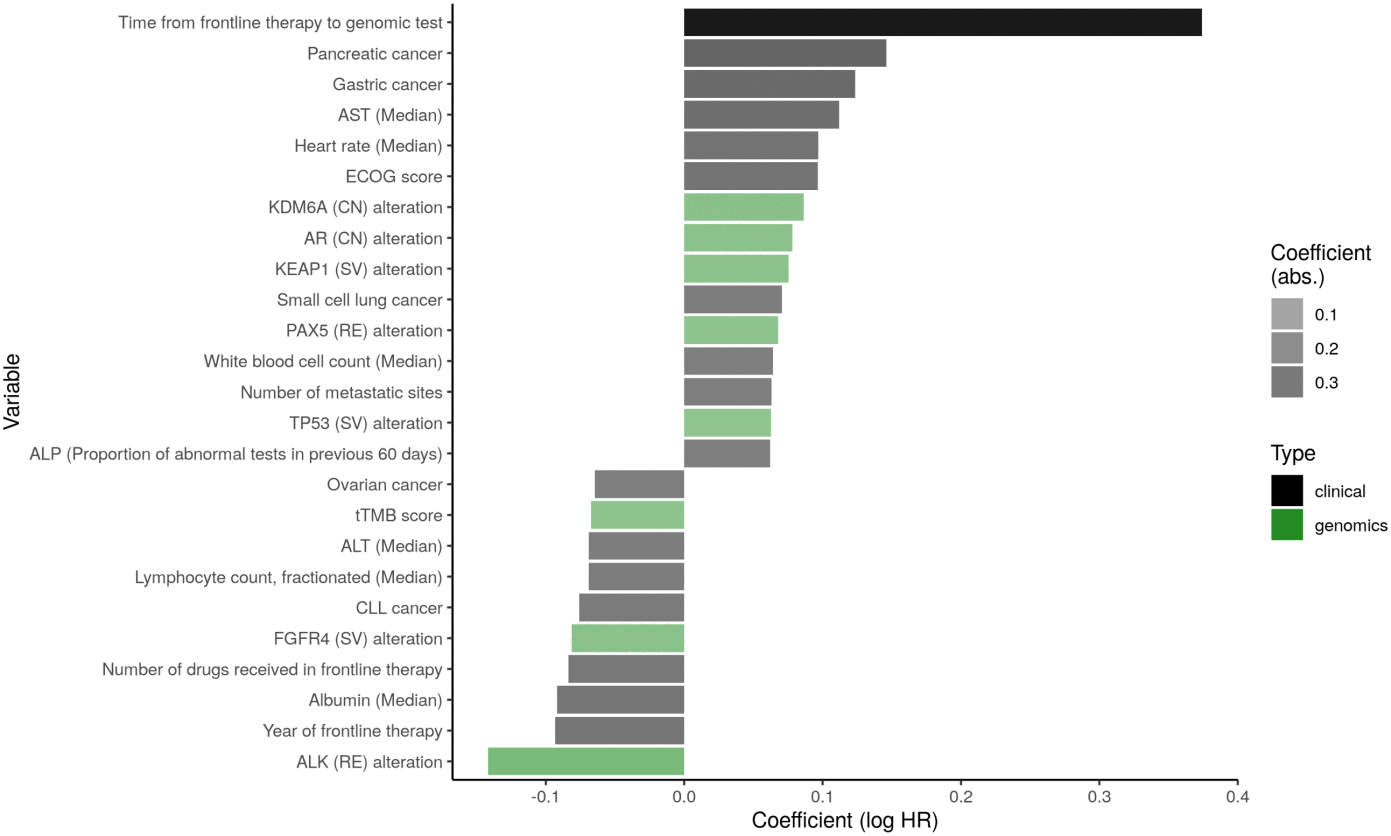
The top 25 predictors selected by the full penalized pan-cancer model, ordered and shaded by coefficient size (log hazard ratio, HR) and colored by predictor type (black = clinical, green = genomics). Positive coefficients suggest a more harmful association with survival and negative coefficients suggest a more favorable association. AST: Aspartate Aminotransferase; ALP: Alkaline Phosphatase; tTMB: tissue Tumor Mutational Burden; ALT: Alanine Transaminase.

Our high-dimensional pan- and single-cancer models offer the opportunity to assess variables strongly associated with cancer prognosis, out of all variables available in the clinico-genomic database. Our penalized approach using lasso regularization provides feature selection by shrinking the coefficients of unimportant variables to zero and retaining only the most prognostic features in the model. Of all 2,059 predictors (2,135 after one-hot encoding), the pan-cancer full model selected a total of 354 (described in Table S3 in S2 File). Fig 4 shows the coefficients of the top 25 pan-cancer predictors, interpreted as the log hazard ratio (HR) where positive coefficients indicate worse prognosis (harmful association with survival) and negative coefficients indicate better prognosis (favorable association with survival). Several of these predictors were described in the previous section as having contributed to the effective pan-cancer risk stratification (Fig 3A). Notably, the clinical features associated with substantially worse survival (log HR > 0.09) were longer time from frontline therapy to genomic test, pancreatic and gastric cancer types, higher ECOG score, higher aspartate aminotransferase (AST) levels, and higher heart rate. Clinical features associated with substantially better survival (log HR < −0.09) were more recent year of frontline treatment and higher albumin levels. Indicators for cancer type suggested pronounced differences in cancer-specific survival: gastric and pancreatic were strongly associated with worse survival, whereas CLL, a slow-growing blood cancer, was associated with longer survival. Seven genomic mutations were among the top 25 pan-cancer predictors overall: KDM6A (CN), AR (CN), KEAP1 (SV), PAX5 (RE), and TP53 (SV) (all associated with worse survival), and FGFR4 (SV) and ALK (RE) (both associated with better survival). In addition, higher tissue tumor mutational burden (tTMB) was associated with better survival.

Our single-cancer models, trained on the equivalent feature set of over 2,000 clinico-genomic predictors but learning from only patients of a single cancer type, offered insights into features important in each cancer setting independently. In contrast with the pan-cancer model, genomic features were more commonly selected as top predictors in the single-cancer models and revealed unique cancer-specific genomic profiles with mutations or cancer pathways not identified as top predictors in the pan-cancer model. Figs S7–S9 in S1 File show these top predictors for each of the 16 cancer types assessed. While a vast majority (89.5%) of patients were diagnosed with advanced or metastatic disease by 1L initiation, the prognostic effect of advanced or metastatic disease was observed in certain cancers with a mix of early- and advanced-stage patients. For example, in ovarian cancer, 32.4% of patients were diagnosed with advanced/metastatic disease, and this variable was associated with significantly worse survival outcomes (Fig S7D in S1 File).

**Fig 5:**
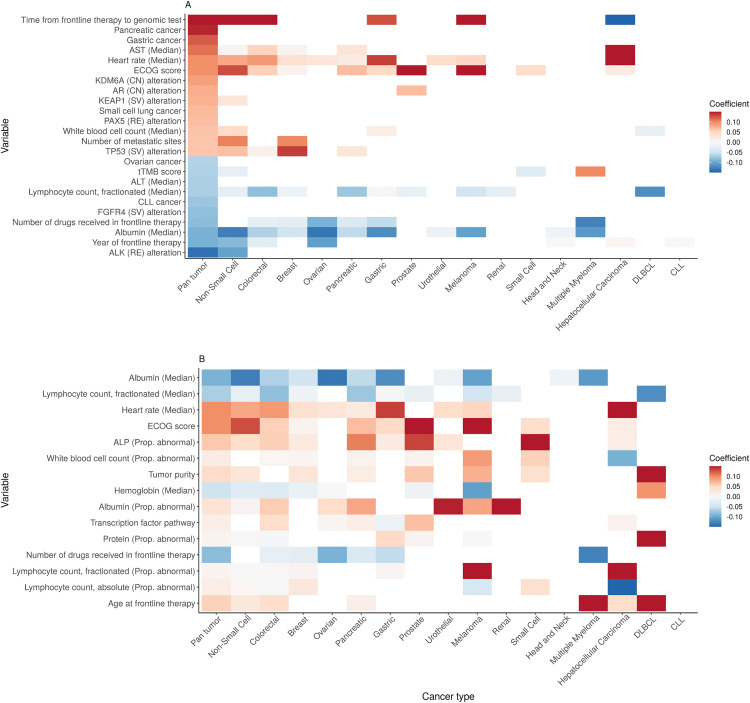
Heatmaps showing the coefficients, by cancer setting, of (A) the top 25 pan-cancer variables and (B) the 15 variables that were selected by at least 7 single-cancer models. In (A), variables on the y-axis are arranged by descending coefficient in the pan-cancer model (high to low); in (B), by descending frequency of selection in the single-cancer models (most to fewest). Variables that were not selected in a particular cancer setting are represented as blank (white) tiles. Cancer types are arranged on the x-axis from largest to smallest sample size. AST: Aspartate Aminotransferase; ALP: Alkaline Phosphatase; tTMB: tissue Tumor Mutational Burden; ALT: Alanine Transaminase; Prop: Proportion.

In addition, evaluating the clinical and genomic predictors selected by multiple single-cancer models could reveal relationships between cancer types and corroborate findings of the pan-cancer model. Fig 5 shows the coefficients of (A) the top 25 pan-cancer variables and (B) the 15 clinico-genomic variables that were selected by at least 7 pan- and single-cancer models. Strong consistency in effect size and direction was observed across several cancers, including for the genomic variables presence of a TP53 (SV) mutation and higher tumor purity (both associated with worse survival); and for the clinical variables older age, higher ECOG score, higher heart rate, and higher proportion of abnormal results for lab tests like alkaline phosphatase (ALP), albumin, and lymphocyte count (all associated with worse survival). Across most cancer cohorts, there was also consistency in the effects of 2 temporal variables: year of frontline therapy (more recent years are associated with better survival) and time from frontline treatment to genomic test (longer interval is associated with worse survival). The variable “time from frontline treatment to genomic test” was derived as the duration from initiation of the patient’s first systemic treatment and the date of the genomic test, reflecting the time from start of therapy to genomic profiling (“entry time”). This variable captures when in the treatment course genomic testing occurred, which can influence observed outcomes if testing is performed later in the disease trajectory.

Most interesting, perhaps, are the predictors selected exclusively by the pan-cancer model and which appear in the model’s top 25 variables (Fig 5A): the mutations in KDM6A (CN), PAX5 (RE), and FGFR4 (SV). These mutations all exhibited stronger association with survival compared to TP53 (SV, the most frequent mutation across all patients), with log HRs between 0.07–0.14 in absolute value, yet were not selected by any cancer-specific model.

Finally, we note that the sparsity of the single-cancer models — how many variables were selected in each cancer cohort — is related to their sample size. Because variables are shrunken by the model to prevent overfitting, the smallest cancer subgroups are often constrained from selecting as many variables as the larger subgroups (Fig S10 in S1 File). As a result, the present feature analysis considers a limited interpretation of variable selection: variables selected by models are considered informative, but the absence of variables is not necessarily informative and may be instead a consequence of sample size.

## Discussion

Our systematic analysis of prognostic pan-cancer and single-cancer models demonstrates the value of a comprehensive framework for prognostic modeling, namely around: model performance tradeoffs, shared cancer biologies, and novel pan-cancer predictors.

First, we observed that, compared to single-cancer models, pan-cancer models demonstrated improved performance and risk stratification capabilities in many cancer types, specifically when the sample size and event rate was small and the training set had a large number of predictors. In the highest-dimensional models (the “full” model), the ability to train on an extensive number of clinico-genomic factors across multiple cancer types was a learning advantage of the pan-cancer model, allowing it to select a vast number of predictors compared to the equivalent single-cancer model, which could be considered an example of transfer learning in low-data settings [17]. Transfer learning assumes that predictive features learned from training in some domain can be applied to a different domain – in this case, cancer types. In our analysis, this phenomenon more accurately reflects a gain in statistical efficiency and the ability to capture shared structure across related cancers, rather than formal transfer learning achieved through pre-training and fine-tuning. This is apparent when studying the performance differences between the pan-cancer and single-cancer models. For the largest cancer cohorts (n > 1,000 patients) like NSCLC and breast cancer, little difference was made because both the pan-cancer and single-cancer datasets were sufficiently large to train on many relevant predictors. For example, in NSCLC (n > 5,000 patients), the single-cancer model selected 115 variables compared to the pan-cancer model’s selection of 354. Moreover, the large cancer single-cancer models shared many of the same predictors selected by the pan-cancer model, such as metastatic sites, ALK mutation (specific to NSCLC), and estrogen receptor (ER) positive status (specific to breast cancer). However, for the smallest cancer cohorts, the models were heavily penalized resulting in the tendency to select fewer features. For head and neck cancer (n = 319 patients), the single-cancer model selected only 6 variables out of > 2,000, but its performance markedly improved with the pan-cancer model which ultimately selected 354 variables. This learning advantage of the pan-cancer model allowed it to capture a wide range of prognostic factors to apply to prediction and risk stratification, especially in smaller cancer settings.

In this context, the observed improvement likely arises from shared feature distributions and increased statistical power gained by pooling patients across cancers, rather than from sequential pre-training and fine-tuning procedures typical of transfer learning. Nonetheless, this pattern suggests that future work could explicitly investigate formal transfer learning approaches to further enhance model generalizability and performance in low-data cancer settings.

However, pan-cancer models did not show a learning advantage in simpler, lower-dimensional settings. In our comparator models (benchmark and ROPRO-like models) which contained a considerably smaller number (< 30) of features, performance was more similar between pan- and single-cancer models. In these scenarios, data from multiple cancer types may not provide substantial additional information or predictive power over what is available in the single cancer setting, particularly since these models already include a small number of known highly prognostic factors such as age, ECOG score, and lab tests. Further, these simple models still perform quite well thanks to the inclusion of ECOG, which was identified by the full (highest-dimensional) model as a top 10 predictor out of > 2,000 features: even the simplest pan-cancer benchmark model, which included ECOG, achieved a c-index of 0.63 (95% CI: 0.62, 0.65) compared to the full model’s c-index of 0.673 (95% CI: 0.667, 0.687). These results suggest that prognostic models developed using a handful of variables collected in routine clinical practice may be sufficient for certain applications, with the benefit of being easier to implement. Indeed, smaller cancer cohorts like Renal Cell Carcinoma (RCC) did not see much performance improvement moving from simple to complex models; however, larger cancer cohorts with > 500 patients like colorectal, prostate, and melanoma saw marked improvements with the inclusion of additional predictors. Here, the decision to use high-dimensional models should consider the disease setting and weigh tradeoffs including: the impact of the performance increase, the feasibility of collecting more data, the desire to include additional clinico-genomic information, and the need for more computationally intensive processes.

Second, our study revealed strong consistency in the predictors identified by both pan-cancer and single-cancer models, underscoring the presence of common clinical and genomic features that contribute to cancer prognosis and corroborating known disease biology. Across all comparator models and across pan-cancer and single-cancer settings, demographic and clinical features like age, ECOG score, and cancer stage at diagnosis were found to be highly prognostic, with older age, higher ECOG score, higher proportion of abnormal lab results, higher AST, and higher heart rate all associated with worse survival outcomes, echoing what is extensively published in the literature and aligning with the findings of the ROPRO model [9,18–22]. Other lab tests like higher albumin levels, higher lymphocyte count, and higher hemoglobin (non-anemic status) were consistently associated with better survival, also aligning with literature and the ROPRO model [23–26]. Briefly, low hemoglobin is a marker of anemia, indicating insufficient oxygen transport to tissues, compromising immune response [27]; low albumin reflects poor nutritional status, impairing the body’s ability to fight cancer and recover from treatment [23]; and low lymphocyte count signifies weakened immune surveillance, reducing the body’s capacity to detect and destroy cancer cells [26]. Certain factors related to care were also associated with better survival outcomes across many cancer types including receiving a higher number of drugs in frontline therapy and being treated in more recent years.

Several genomic factors also exhibited strong consistency across cancer types. Higher tumor purity was consistently associated with worse survival in 7 cancer types. The concept of tumor purity refers to the extent to which the tumor tissue consists of cancer cells versus other types of cells present in the tumor microenvironment; a higher tumor purity indicates a larger proportion of cancer cells relative to non-cancerous cells, and here is measured in silico (using epigenomic, genomic, or transcriptomic profiles) [28]. Multiple factors contribute to the tumor purity estimate, including ease of sampling and specific tissue of origin, but an intriguing hypothesis that could explain the effect observed in these data is immune infiltration. The immune system plays a crucial role in recognizing and eliminating abnormal or cancerous cells, but when tumor purity is high, this functionality is inhibited. Another possibility is that samples with higher tumor purity are representative of larger or more aggressive tumors with poor survival. It is possible that tumor samples with higher purity are preferentially selected for testing, although if so, we expect this limitation to be consistent across all samples, suggesting the baseline tumor purity values are likely systematically higher rather than introducing directional bias. Thus, the finding that high tumor purity is a strong predictor of worse survival is likely aligned with our understanding of cancer biology but may be reflective of the specific sample rather than the whole tumor [29]. Moreover, the TP53 (SV) mutation was associated with worse survival in 5 cancer settings, consistent with the well-established role of TP53 in tumor suppression and its implications in cancer progression [30].

A new finding was the strong effect of time from frontline therapy to genomic test, across cancers. As mentioned, this variable reflects the time from start of therapy to genomic profiling and captures when in the treatment course genomic testing occurred. Importantly, it is a marker of selection bias – since patients must survive long enough to undergo genomic testing. The left truncation-adjusted models adjusted for this variable to achieve quasi-independence between database entry time (marked by receiving a genomic test) and survival time and correct for this bias, described in Materials & Methods. This feature is an important phenomenon in the data, where many patients receive genomic tests long after frontline treatment, and could have implications for patient outcomes. The strength of this association indicates some residual bias may remain, for example if the likelihood of testing is related to unmeasured clinical factors or care pathways. One hypothesis to explain its strong association is that patients who receive genomic tests later in their treatment course may have exhausted standard treatment options. As a result, they may be considered high-risk with limited therapeutic options. This finding raises some considerations for clinical practice: earlier genomic testing in the treatment course may provide more personalized treatment options (e.g., biomarker targeted therapy) for patients, potentially leading to better outcomes. Further, the time interval between diagnosis and genomic test is shrinking over time thanks to the increased availability and access to genomic testing in recent years, so this association may weaken in the future.

Finally, our analysis uncovered potentially unique pan-cancer variables. The somatic mutations in KDM6A (CN), PAX5 (RE), and FGFR4 (SV), identified among the top 25 features of the pan-cancer model, had similar prevalence across multiple cancer types but were not selected by any of the single-cancer models and thus warrant further research. PAX5 may be implicated in metastasis [31], KDM6A in DNA damage repair, and the FGFR family of proteins in a number of cell proliferation pathways [32]. These may have multi-cancer relevance. Alterations in KDM6A have been observed across a broad range of tumor types, including bladder, pancreatic, renal, and hematologic malignancies, and are frequently associated with loss of tumor-suppressive demethylase activity and poorer prognosis, supporting a potential pan-cancer relevance of this gene [33]. Similarly, dysregulation of FGFR4 and the broader FGFR signaling axis has been linked to aggressive tumor behavior, with FGFR inhibitors already being evaluated in diverse cancer settings [34]. PAX5, classically involved in B-cell lineage specification, has also been shown to be expressed or exert context-dependent oncogenic or tumor-suppressive effects in several non-hematologic cancers [35,36]. Importantly, however, we recognise that the prognostic associations of these genes may be confounded by treatment regimens or subtype-specific therapeutic targeting: for instance, FGFR pathway inhibitors are already in clinical use in some FGFR-altered cancers, which could influence survival associations (FGFR has a protective association with survival in our study). These findings therefore highlight intriguing biological hypotheses that warrant mechanistic investigation and validation in external datasets and experimental models.

While not the focus of this paper, our study also reveals the unique clinico-genomic profile of each cancer type by showcasing the top 25 features of each single-cancer model, which can be explored in Figs S7–S9 in S1 File.

Prognostic models hold significant utility in oncology research and clinical practice. Better risk stratification on the basis of prognosis can help physicians and clinical trials identify high-risk patients who may benefit from more intensive interventions or personalized treatment strategies; conversely, they can also spare low-risk patients from unnecessary interventions and thus help optimize resource allocation. In many cancer settings, we observed that pan-cancer models improved risk stratification because of their training advantages discussed previously. Separately, our models also corroborate the good discriminative ability of the ROPRO prognostic model in both pan- and single-cancer settings, which has demonstrated clinical utility in several applications [9,37].

Our prognostic modeling framework is a strength of this work that enables a consistent evaluation of multiple models in diverse pan- and single-cancer settings. It can be used as a template to extend this study to future research areas, including: further work on the theory behind “low information” transfer learning approaches like the one demonstrated in this study; the concept of “pre-training” to give advantage in low information settings; and exploring cancer subgroups (such as hematological or hormone-dependent) within which similarities can be further exploited by this “pan” training approach.

Our study has several limitations that should be considered when interpreting the results. The sample sizes and number of events in many cancer types were relatively small, which can lead to model instability and imprecise estimates, as indicated by the wide confidence intervals for the smallest cancer cohorts like DLBCL (n = 122) and CLL (n = 83) as well as unstable coefficient sizes and very sparse models. As a result, findings that compare model performance remain trends and are descriptive. Additionally, the analysis used penalized linear Cox models, which assume a linear relationship between predictors and the log-hazard ratio. While this is a commonly used approach, it may not capture potential non-linear associations between predictors and outcomes nor complex interactions between variables. For example, certain clinical parameters, such as ECOG score or treatment type (e.g., biomarker-targeted therapy), have known effects on survival that could confound the interpretation of genomic effects. To investigate whether the ability to model complex and nonlinear associations and interactions would improve performance, we implemented a tree-based approach in the form of random survival forests adjusted for left truncation [38] and found no difference in performance compared to our linear models (Fig S11, SI Materials and Methods in S1 File), which suggests the data may be adequately modeled using linearity assumptions, which also yield more interpretable results (hazard ratios) for clinical audiences. While RSF captures such interactions, it does not offer substantial predictive improvement. Therefore, the interpretation of individual genomic variable effects should be approached with caution, as their prognostic contributions may not be fully independent of clinical parameters. Additionally, proportional hazards (PH) diagnostics were not formally assessed, and potential deviations from the PH assumption could influence the interpretation of hazard ratio estimates. Our models adjusted for left truncation, a feature of the clinico-genomic database, but this could introduce bias if the truncation is dependent on the outcome. Further, high levels of missingness in the clinico-genomic database led us to omit potential prognostic factors like lactate dehydrogenase (LDH), preventing us from perfectly replicating the ROPRO model [9] and potentially impacting the comprehensiveness of the models.

It is also important to note that our models did not include interaction effects of mutations with treatment and so did not explicitly model predictive biomarkers [39]. For example, in the case of NSCLC, ALK mutations were shown to be protective, likely due to the availability of ALK inhibitors as approved treatments. This illustrates the distinction between a prognostic biomarker – one that is associated with outcome regardless of therapy – and a predictive biomarker, whose effect depends on treatment exposure. Because our models do not include explicit interaction terms between genomic features and treatment type, mutation coefficients should be interpreted as marginal prognostic associations rather than treatment-modified effects. In real-world datasets such as ours, heterogeneity in treatment regimens (e.g., varying use of targeted therapies) can confound survival associations and influence model predictions. Future analyses could address this by stratifying on treatment class or incorporating mutation-treatment interaction terms within models. The absence of such interaction effects in our models limits the interpretation of the predictive value of specific mutations in the context of treatment response, and this is explored in other studies [40]. For example, Liu et al. (2022) conducted a large pan-cancer survival analysis including *explicit* mutation-treatment interactions using the same real-world clinico-genomic data, demonstrating how specific alterations such as EGFR, ALK, and BRAF mutations can modify therapeutic response patterns across cancers.

A final limitation of this study is the reliance on a single dataset, albeit the largest commercially available and comprehensive clinico-genomic dataset of its kind. While this dataset offers unique scale and granularity for pan-cancer prognostic modeling, the absence of an external validation cohort limits the generalizability of our findings. Future work should prioritize external validation using publicly available resources such as The Cancer Genome Atlas (TCGA), the AACR GENIE consortium, or other large-scale clinico-genomic cohorts. However, applying our models to these datasets will require careful preprocessing and feature harmonization. For example, TCGA and AACR GENIE provide extensive genomic and limited clinical data but may lack variables such as comorbidity indices, detailed treatment histories, or longitudinal laboratory measures that were incorporated in our models. These datasets could therefore serve as valuable benchmarks for validating the “benchmark” and “ROPRO-like” models that rely primarily on demographic and performance features, whereas the higher-dimensional clinico-genomic models may require adaptation to a reduced set of harmonized predictors. Replicating this “pan-cancer vs. single-cancer” hypothesis in external datasets, particularly in distinct clinical settings or patient populations, would be a valuable next step to confirm the robustness and broader applicability of these models. We encourage the scientific community to test this approach in other datasets to further validate its utility and potential.

Despite these limitations, our study offers a comprehensive, large-scale, and data-driven assessment of cancer that may be valuable for hypothesis generation, prognostic modeling, and risk stratification in oncology.

## Materials and methods

### Data

Patient-level data and outcomes were derived from the pan-tumor CGDB offered jointly by Flatiron Health and Foundation Medicine. The CGDB is a US nationwide, longitudinal, de-identified oncology database that combines real-world, patient-level clinical data and outcomes with patient-level genomic data from over 280 US cancer clinics (approximately 800 sites of care). Comprehensive genomic profiling of >300 cancer-related genes on Foundation Medicine next-generation sequencing tests (including both current solid and liquid assays and legacy assays: FoundationOne CDx, FoundationOne Liquid CDx, FoundationOne Heme, FoundationOne, FoundationOne Liquid, FoundationACT) were linked to Flatiron EHR patient data via de-identified, deterministic matching [41–43]. To date, over 400,000 samples have been sequenced from patients across the US. The data are de-identified and subject to obligations to prevent reidentification and protect patient confidentiality. Altogether, the CGDB contains a rich set of thousands of potentially important prognostic factors for survival, including demographic characteristics, treatment regimens, disease and diagnosis profiles, mutational status of cancer-related genes, and longitudinal records of laboratory tests.

Patients from 16 cancer cohorts with a recorded oncology clinician-defined, rule-based, first line of therapy (1L) between January 1, 2011 through June 30, 2020 were pooled into a single, pan-cancer cohort containing 28,079 patients, with data originally accessed for research on August 31, 2021. The pan-cancer cohort comprised the following 16 cancer types: breast, chronic lymphocytic leukemia (CLL), colorectal, diffuse large B-cell lymphoma (DLBCL), gastric, head and neck, hepatocellular carcinoma (HCC), melanoma, multiple myeloma, non-small cell lung cancer (NSCLC), ovarian, pancreatic, prostate, renal, small-cell lung cancer (SCLC), and urothelial. Patients were split into a train set (80%) and test set (20%) using stratified random sampling by cancer type.

### Feature engineering and preprocessing

All available data from the CGDB were used to derive a suite of features for each patient corresponding to 5 data modalities: clinical/demographic, laboratory/vital signs, treatment, cancer-specific (these 4 collectively referred to as “clinical”), and genomic. These features are summarized at in Table S1 in S1 File. Features were eliminated in a data-driven way if they were zero-variance, near-zero-variance (dummy variables with fewer than 20 counts), or missing in over 30% of the pan-cancer cohort. Multiple imputation by chained equations (MICE) was used to impute clinical features and a k-nearest neighbors (kNN) approach [44] was used to impute genomic features incorporating 2,566 samples from The Cancer Genome Atlas (TCGA) that had information on mutations of all three relevant types available: short variants (SNVs, indels), copy number alterations (CN), and rearrangements (fusions, RE) [45]. Since the TCGA data were derived from whole genome sequencing, we filtered the data to only those genes measured on Foundation Medicine panels. All imputation was performed in the train set separately from the test set to generate m = 5 imputed datasets. Because of complex pooling, the results are presented for the first imputed dataset (m = 1) and results were subjectively similar across imputed datasets. Specific feature engineering efforts are described below.

Clinical-demographic information included information on patient age, gender, race, smoking status, body mass index (BMI), cancer type, cancer stage at diagnosis, advanced or metastatic status of the cancer at baseline, Eastern Cooperative Oncology Group (ECOG) Performance Status, and a composite measure of comorbidity (the Elixhauser comorbidity index [46,47]) derived from structured EHR diagnosis code data. Treatment was represented in the form of indicators for the unique drug category (e.g., chemotherapy, immunotherapy, targeted/biologic, targeted/nonbiologic) received during the first line of therapy (1L). The number of unique drugs received in 1L, year of frontline therapy, time from diagnosis to first treatment, and treatment at an academic center (vs. community center) were also included. In addition, the variable “time from frontline therapy to genomic test” was derived as the interval between initiation of the patient’s first systemic treatment and the date of the Foundation Medicine assay, reflecting the time from start of therapy to genomic profiling (“entry time”). This variable captures when in the treatment course genomic testing occurred, which can influence observed outcomes if testing is performed later in the disease trajectory.

Time series summaries of over 100 longitudinal laboratory tests and vital signs were computed within 2 time windows prior to the patient’s first line of therapy initiation date: 60 days (~2 months) and 720 days (~2 years). The following metrics were computed within each window: mean, median, variance, max, min, approximate entropy, difference between the last 2 values, slope of the last 2 values, total number of tests, ratio of number of tests to the available window of data observed for each patient, and, for lab tests, the proportion of labs that were abnormal. For comparability across patients and testing devices, lab values were normalized to their upper and lower limits of normal. Clinical input was obtained to assign thresholds of plausible lab and vital sign values; outlying values were set to missing and imputed.

Genomic features were generated from a single specimen per patient, choosing the specimen collected closest to index date if multiple were available. Binary indicator variables were populated from the mutations assessed by the specimen’s Foundation Medicine panel, coding each gene’s short variant, copy number, and rearrangement status separately. Gene-variant combinations that were not measured on a panel were coded as “NA” and were later imputed in the k nearest neighbors step. To summarize the alterations on a pathway level, we used a literature-derived list of gene sets and coded a pathway as impacted if any of its constituent genes had a reported mutation. Another feature type that introduced external information was the node2vec derived values, which were derived by averaging the node2vec [48] embeddings vector of all affected genes in a specimen. The embeddings themselves were computed by running the reference implementation of the node2vec algorithm (https://github.com/aditya-grover/node2vec) with default settings using as input the human protein-protein interaction network available from the HitPredict effort [49,50]. For interpretability, Fig S12 in S1 File presents the genomic variable contributions to the key protein interaction networks (“node2vec”) selected in the pan- and single-tumor models. A final set of features incorporating the observed mutation statuses and external data were the computed exposures to previously published mutational signatures [51]. These exposures were inferred via the SigsPack R package [52]. While the majority of specimen-derived features harnessed the Foundation Medicine mutation readout, a handful of standalone scores were also incorporated – tumor mutational burden, tumor purity, PDL1 status, estimated percentage of genome loss of heterozygosity, and microsatellite instability status.

Finally, cancer-specific features were obtained from records unique to each of the 16 cancer cohorts. These were included for their potential importance in predicting cancer-specific survival. Examples include the Gleason Score (a prognostic grading score for patients with prostate cancer), metastatic sites (for metastatic cancers such as breast cancer and non-small cell lung cancer), and transformation status (denoting transformation from follicular lymphoma to diffuse large b-cell lymphoma). These cancer-specific features exhibit “structured missingness [53]” in the pan-cancer cohort: available for one or few cancer types, and missing for the rest. As a result, they were not imputed outside of the relevant cancer cohort(s) and instead were set to 0. These variables are listed in Table S4 in S1 File.

Categorical variables were one-hot encoded with the reference level set to the majority level. All variables were normalized and outliers were truncated at +/- 3 z-scores for model stability.

### Model development

A penalized Cox proportional hazards model with lasso regularization was used to predict overall survival (OS) in the pan-cancer cohort using 2,059 (2,135 after one-hot encoding) CGDB-derived features. Survival time was calculated from 1L initiation date to death or last activity record in the EHR. Note that Flatiron EHR mortality records are validated against the National Death Index (NDI), widely considered a gold standard death dataset in the US, and shown to have high sensitivity, specificity, and date accuracy. A risk set adjustment was used to adjust for left truncation (see McGough *et al*. [54] for a discussion on left truncation in this data source), and the model was adjusted for entry time to achieve quasi-independence between entry time and survival time [55]. Additionally, the model was adjusted for cancer type and compared to a stratified Cox model to account for potentially different baseline hazards by cancer type. Stratified Cox models were similar to but slightly outperformed by the non-stratified models (Fig S13 in S1 File) and so are not described in the main text. Thus the main text describes pan-cancer models adjusted for cancer type. All models were fit using glmnet v. 4.0 [56,57] which handles left-truncated and right-censored survival data.

Patients were split into a train set (80%) and test set (20%) using stratified random sampling by cancer type. Five-fold cross-validation was used to tune the penalized model and the value of the lasso penalty, λ, that maximized the concordance index was selected to give the final model. Out-of-sample pan-cancer predictions were made on the withheld test set comprising (i) the overall pan-cancer cohort and (ii) each single-cancer cohort.

To compare predictions and insights gained from pan-cancer settings to those gained from single-cancer settings, a series of equivalent single-cancer models were developed dynamically using the original feature set. Feature normalization, detection and removal of zero- and near-zero-variance predictors, and truncation of outliers were performed separately in each single-cancer cohort, driven by the available data for that cancer type to simulate a real-world single-cancer setting.

Pan- and single-cancer models constructed on the full CGDB data were benchmarked against simpler models from clinical practice and the literature: (1) a benchmark model containing cancer type, age, gender, race, smoking status, cancer stage at diagnosis, baseline Eastern Cooperative Oncology Group (ECOG) Performance Status, time from diagnosis to initiation of 1L, and time from genomic test to initiation of 1L; and (2) a model adapted from ROPRO (Real wOrld PROgnostic score) by Becker et al. [9]. These models are described in Table S2 and SI Materials & Methods in S1 File.

### Model evaluation

Pan- and single-cancer models were evaluated using the out-of-sample concordance index (c-index) and integrated Brier score (IBS). Additionally, predicted risk scores were calculated for each patient as the exponential of the linear predictors from the penalized Cox model. Predicted risk scores were then used to stratify test set patients into high- and low-risk categories in each cancer cohort based on the median risk score of the training patients in the cohort.

Bias-corrected bootstrap percentile intervals [16] were used to quantify uncertainty in model performance metrics using B = 1,000 bootstraps of the train and test data.

### Software

All analyses were performed using R v. 4.1.1 (R Core Team 2021) [58].

Data ingestion, manipulation, and preprocessing was performed using the R packages dplyr (v1.0.7) [59], dbplyr (v2.1.1) [60], rlang (v1.1.0) [61], data.table (v1.14.0) [62], tidyr (v1.1.3) [63], stats [58], purrr (v1.0.1) [64], wrapr (v2.0.8) [65], stringr (v1.4.0) [66], hashmap (v0.2.2) [67], pracma (v2.3.3) [68], rsample (v0.1.0) [69], fastDummies (v1.6.3) [70], and coder (v0.13.5) [71]. Data imputation was performed using mice (v3.13.0) [72] and impute (v1.65.0) [73], and models were run using glmnet (v4.1-3) [56], survival (v3.2-13) [74], caret (v6.0-88) [75], and LTRCforests (v0.5.5) [76,77]. Code parallelization and execution was performed using doParallel (v1.0.16) [78], foreach (v1.5.1) [79], doFuture (v0.12.0) [80], parallel [58], rngtools (v1.5) [81], and doRNG (v1.8.2) [82] and logged using logger (v0.2.1) [83]. Finally, figures were rendered using ggplot2 (v3.3.5) [84].

### Ethics, consent, and permissions

We confirm all relevant ethical guidelines have been followed, and any necessary IRB and/or ethics committee approvals have been obtained.

As this is an observational study that uses de-identified data per expert determination that was previously collected, this study does not constitute human subjects research under the Common Rule and thus did not require IRB oversight or patient informed consent.

We confirm that the appropriate institutional forms have been archived, and that any patient/participant/sample identifiers included were not known to anyone and cannot be used to identify individuals.

## Supporting information

S1 File The Supporting information file contains SI Tables 1–2, SI Figures 1–13, and SI Materials & Methods. Table S3 is provided separately as an Excel file.
(DOCX)

S2 File Table S3. List of the 354 variables selected by the pan-cancer “full” model. (CSV)

## References

[1] Kattan MW, Hess KR, Amin MB, Lu Y, Moons KGM, Gershenwald JE, et al. American Joint Committee on Cancer acceptance criteria for inclusion of risk models for individualized prognosis in the practice of precision medicine. CA Cancer J Clin. 2016;66(5):370–4. doi: 10.3322/caac.21339 26784705 PMC4955656

[2] Viele K, Girard TD. Risk, results, and costs: optimizing clinical trial efficiency through prognostic enrichment. Am J Respir Crit Care Med. 2021;203(6):671–2. 33075242 10.1164/rccm.202009-3649ED PMC7958522

[3] Simon R. Clinical trial designs for evaluating the medical utility of prognostic and predictive biomarkers in oncology. Per Med. 2010;7(1):33–47. 20383292 10.2217/pme.09.49 PMC2851173

[4] International Non-Hodgkin’s Lymphoma Prognostic Factors Project. A predictive model for aggressive non-Hodgkin’s lymphoma. N Engl J Med. 1993;329(14):987–94. 8141877 10.1056/NEJM199309303291402

[5] Jang RW, Caraiscos VB, Swami N, Banerjee S, Mak E, Kaya E, et al. Simple prognostic model for patients with advanced cancer based on performance status. J Oncol Pract. 2014;10(5):e335-41. doi: 10.1200/JOP.2014.001457 25118208

[6] Dhiman P, Ma J, Andaur Navarro CL, Speich B, Bullock G, Damen JAA, et al. Methodological conduct of prognostic prediction models developed using machine learning in oncology: a systematic review. BMC Med Res Methodol. 2022;22(1):101. doi: 10.1186/s12874-022-01577-x 35395724 PMC8991704

[7] Marabelle A, Fakih M, Lopez J, Shah M, Shapira-Frommer R, Nakagawa K, et al. Association of tumour mutational burden with outcomes in patients with advanced solid tumours treated with pembrolizumab: prospective biomarker analysis of the multicohort, open-label, phase 2 KEYNOTE-158 study. Lancet Oncol. 2020;21(10):1353–65. doi: 10.1016/S1470-2045(20)30445-9 32919526

[8] Doebele RC, Drilon A, Paz-Ares L, Siena S, Shaw AT, Farago AF, et al. Entrectinib in patients with advanced or metastatic NTRK fusion-positive solid tumours: integrated analysis of three phase 1-2 trials. Lancet Oncol. 2020;21(2):271–82. doi: 10.1016/S1470-2045(19)30691-6 31838007 PMC7461630

[9] Becker T, Weberpals J, Jegg AM, So WV, Fischer A, Weisser M, et al. An enhanced prognostic score for overall survival of patients with cancer derived from a large real-world cohort. Ann Oncol. 2020;31(11):1561–8. doi: 10.1016/j.annonc.2020.07.013 32739409

[10] Halabi S. Pan-cancer prognostic models of clinical outcomes: statistical exercise or clinical tools? Ann Oncol. 2020;31(11):1427–9. doi: 10.1016/j.annonc.2020.08.2233 32891792

[11] Julian C, Machado RJM, Girish S, Chanu P, Heinzmann D, Harbron C, et al. Real-world data prognostic model of overall survival in patients with advanced NSCLC receiving anti-PD-1/PD-L1 immune checkpoint inhibitors as second-line monotherapy. Cancer Rep (Hoboken). 2022;5(10):e1578. doi: 10.1002/cnr2.1578 35075804 PMC9575492

[12] Kratz JR, Jablons DM. Genomic prognostic models in early-stage lung cancer. Clin Lung Cancer. 2009;10(3):151–7. doi: 10.3816/CLC.2009.n.021 19443334

[13] Fan C, Prat A, Parker JS, Liu Y, Carey LA, Troester MA, et al. Building prognostic models for breast cancer patients using clinical variables and hundreds of gene expression signatures. BMC Med Genomics. 2011;4:3. doi: 10.1186/1755-8794-4-3 21214954 PMC3025826

[14] Singal G, Miller PG, Agarwala V, He J, Gossai A, Frank S. Development and validation of a real-world clinicogenomic database. J Clin Oncol. 2017;35(15_suppl):2514.

[15] Birnbaum B, Nussbaum N, Seidl-Rathkopf K, Agrawal M, Estevez M, Estola E, et al. Model-assisted cohort selection with bias analysis for generating large-scale cohorts from the EHR for oncology research [Internet]. 2020 [cited 2023 Oct 9]. Available from: http://arxiv.org/abs/2001.09765

[16] Efron B, Tibshirani R. Bootstrap methods for standard errors, confidence intervals, and other measures of statistical accuracy. Stat Sci. 1986;1(1):54–75.

[17] Roster K, Connaughton C, Rodrigues FA. Forecasting new diseases in low-data settings using transfer learning. Chaos Solitons Fractals. 2022;161:112306. doi: 10.1016/j.chaos.2022.112306 35765601 PMC9222348

[18] Oken MM, Creech RH, Tormey DC, Horton J, Davis TE, McFadden ET, et al. Toxicity and response criteria of the Eastern Cooperative Oncology Group. Am J Clin Oncol. 1982;5(6):649–55. doi: 10.1097/00000421-198212000-00014 7165009

[19] Blagden SP, Charman SC, Sharples LD, Magee LRA, Gilligan D. Performance status score: do patients and their oncologists agree? Br J Cancer. 2003;89(6):1022–7. doi: 10.1038/sj.bjc.6601231 12966419 PMC2376959

[20] Høst H, Lund E. Age as a prognostic factor in breast cancer. Cancer. 1986;57(11):2217–21. doi: 10.1002/1097-0142(19860601)57:11<2217::aid-cncr2820571124>3.0.co;2-t 3697919

[21] Tas F, Ciftci R, Kilic L, Karabulut S. Age is a prognostic factor affecting survival in lung cancer patients. Oncol Lett. 2013;6(5):1507–13. 24179550 10.3892/ol.2013.1566 PMC3813578

[22] Brierley JD, Srigley JR, Yurcan M, Li B, Rahal R, Ross J, et al. The value of collecting population-based cancer stage data to support decision-making at organizational, regional and population levels. Healthc Q. 2013;16(3):27–33. doi: 10.12927/hcq.2013.23497 24034774

[23] Gupta D, Lis CG. Pretreatment serum albumin as a predictor of cancer survival: a systematic review of the epidemiological literature. Nutr J. 2010;9:69. 21176210 10.1186/1475-2891-9-69 PMC3019132

[24] Gou M, Zhang Y, Liu T, Qu T, Si H, Wang Z. The prognostic value of pre-treatment hemoglobin (Hb) in patients with advanced or metastatic gastric cancer treated with immunotherapy. Front Oncol. 2021;11. 10.3389/fonc.2021.655716 PMC8239234 34211839

[25] What is the value of hemoglobin as a prognostic and predictive factor in cancer? Eur J Cancer Suppl. 2004;2(2):11–9.

[26] Zhao J, Huang W, Wu Y, Luo Y, Wu B, Cheng J. Prognostic role of pretreatment blood lymphocyte count in patients with solid tumors: a systematic review and meta-analysis. Cancer Cell Int. 2020. 10.1186/s12935-020-1094-5 PMC6954501 31938023

[27] Caro JJ, Salas M, Ward A, Goss G. Anemia as an independent prognostic factor for survival in patients with cancer: a systemic, quantitative review. Cancer. 2001;91(12):2214–21. doi: 10.1002/1097-0142(20010615)91:12<2214::aid-cncr1251>3.0.co;2-p 11413508

[28] Sun JX, He Y, Sanford E, Montesion M, Frampton GM, Vignot S, et al. A computational approach to distinguish somatic vs. germline origin of genomic alterations from deep sequencing of cancer specimens without a matched normal. PLoS Comput Biol. 2018;14(2):e1005965. doi: 10.1371/journal.pcbi.1005965 29415044 PMC5832436

[29] Aran D, Sirota M, Butte AJ. Systematic pan-cancer analysis of tumour purity. Nat Commun. 2015;6(1):1–12. 10.1038/ncomms9971 PMC4671203 26634437

[30] Petitjean A, Achatz MIW, Borresen-Dale AL, Hainaut P, Olivier M. TP53 mutations in human cancers: functional selection and impact on cancer prognosis and outcomes. Oncogene. 2007;26(15):2157–65. doi: 10.1038/sj.onc.1210302 17401424

[31] O’Brien P, Morin P Jr, Ouellette RJ, Robichaud GA. The Pax-5 gene: a pluripotent regulator of B-cell differentiation and cancer disease. Cancer Res. 2011;71(24):7345–50. doi: 10.1158/0008-5472.CAN-11-1874 22127921

[32] Katoh M. Fibroblast growth factor receptors as treatment targets in clinical oncology. Nat Rev Clin Oncol. 2018;16(2):105–22. 10.1038/s41571-018-0115-y 30367139

[33] Hua C, Chen J, Li S, Zhou J, Fu J, Sun W. KDM6 demethylases and their roles in human cancers. Front Oncol. 2021;11:779918. 34950587 10.3389/fonc.2021.779918 PMC8688854

[34] ClinicalTrials.gov [Internet]. [cited 2025 Nov 24]. Available from: https://clinicaltrials.gov/study/NCT03827850

[35] Mhawech-Fauceglia P, Saxena R, Zhang S, Terracciano L, Sauter G, Chadhuri A, et al. Pax-5 immunoexpression in various types of benign and malignant tumours: a high-throughput tissue microarray analysis. J Clin Pathol. 2007;60(6):709–14. doi: 10.1136/jcp.2006.039917 16837628 PMC1955074

[36] Kanteti R, Nallasura V, Loganathan S, Tretiakova M, Kroll T, Krishnaswamy S, et al. PAX5 is expressed in small-cell lung cancer and positively regulates c-Met transcription. Lab Invest. 2009;89(3):301–14. doi: 10.1038/labinvest.2008.168 19139719 PMC2741690

[37] CN2 ROPRO – Real-World Data Prognostic Score: A Novel Tool to Assess Patients’ Performance Status. Ann Oncol. 2021;32:S1256.

[38] Yao W, Frydman H, Larocque D, Simonoff JS. Ensemble methods for survival function estimation with time-varying covariates. Stat Methods Med Res. 2022;31(11):2217–36. doi: 10.1177/09622802221111549 35895510

[39] Sechidis K, Papangelou K, Metcalfe PD, Svensson D, Weatherall J, Brown G. Distinguishing prognostic and predictive biomarkers: an information theoretic approach. Bioinformatics. 2018;34(19):3365–76. 29726967 10.1093/bioinformatics/bty357 PMC6157098

[40] Liu R, Rizzo S, Waliany S, Garmhausen MR, Pal N, Huang Z, et al. Systematic pan-cancer analysis of mutation-treatment interactions using large real-world clinicogenomics data. Nat Med. 2022;28(8):1656–61. doi: 10.1038/s41591-022-01873-5 35773542

[41] Woodhouse R, Li M, Hughes J, Delfosse D, Skoletsky J, Ma P, et al. Clinical and analytical validation of FoundationOne Liquid CDx, a novel 324-Gene cfDNA-based comprehensive genomic profiling assay for cancers of solid tumor origin. PLoS One. 2020;15(9):e0237802. doi: 10.1371/journal.pone.0237802 32976510 PMC7518588

[42] Frampton GM, Fichtenholtz A, Otto GA, Wang K, Downing SR, He J, et al. Development and validation of a clinical cancer genomic profiling test based on massively parallel DNA sequencing. Nat Biotechnol. 2013;31(11):1023–31. doi: 10.1038/nbt.2696 24142049 PMC5710001

[43] He J, Abdel-Wahab O, Nahas MK, Wang K, Rampal RK, Intlekofer AM, et al. Integrated genomic DNA/RNA profiling of hematologic malignancies in the clinical setting. Blood. 2016;127(24):3004–14. doi: 10.1182/blood-2015-08-664649 26966091 PMC4968346

[44] Troyanskaya O, Cantor M, Sherlock G, Brown P, Hastie T, Tibshirani R, et al. Missing value estimation methods for DNA microarrays. Bioinformatics. 2001;17(6):520–5. doi: 10.1093/bioinformatics/17.6.520 11395428

[45] Hu X, Wang Q, Tang M, Barthel F, Amin S, Yoshihara K, et al. TumorFusions: an integrative resource for cancer-associated transcript fusions. Nucleic Acids Research. 2018;46(D1):D1144–9. 10.1093/nar/gkx1018 PMC5753333 29099951

[46] Elixhauser A, Steiner C, Harris DR, Coffey RM. Comorbidity measures for use with administrative data. Med Care. 1998;36(1):8–27. doi: 10.1097/00005650-199801000-00004 9431328

[47] van Walraven C, Austin PC, Jennings A, Quan H, Forster AJ. A modification of the Elixhauser comorbidity measures into a point system for hospital death using administrative data. Med Care. 2009;47(6):626–33. doi: 10.1097/MLR.0b013e31819432e5 19433995

[48] Grover A, Leskovec J. node2vec: Scalable Feature Learning for Networks. KDD. 2016;2016:855–64. doi: 10.1145/2939672.2939754 27853626 PMC5108654

[49] Patil A, Nakai K, Nakamura H. HitPredict: a database of quality assessed protein-protein interactions in nine species. Nucleic Acids Res. 2011;39(Database issue):D744–9. doi: 10.1093/nar/gkq897 20947562 PMC3013773

[50] López Y, Nakai K, Patil A. HitPredict version 4: comprehensive reliability scoring of physical protein-protein interactions from more than 100 species. Database (Oxford). 2015;2015:bav117. doi: 10.1093/database/bav117 26708988 PMC4691340

[51] Alexandrov LB, Nik-Zainal S, Wedge DC, Aparicio SAJR, Behjati S, Biankin AV, et al. Signatures of mutational processes in human cancer. Nature. 2013;500(7463):415–21. doi: 10.1038/nature12477 23945592 PMC3776390

[52] Schumann F, Blanc E, Messerschmidt C, Blankenstein T, Busse A, Beule D. SigsPack, a package for cancer mutational signatures. BMC Bioinformatics. 2019;20(1):450. doi: 10.1186/s12859-019-3043-7 31477009 PMC6720940

[53] Mitra R, McGough SF, Chakraborti T, Holmes C, Copping R, Hagenbuch N, et al. Learning from data with structured missingness. Nat Mach Intell. 2023;5(1):13–23. doi: 10.1038/s42256-022-00596-z

[54] McGough SF, Incerti D, Lyalina S, Copping R, Narasimhan B, Tibshirani R. Penalized regression for left-truncated and right-censored survival data. Stat Med. 2021;40(25):5487–500. doi: 10.1002/sim.9136 34302373 PMC9290657

[55] Gail MH, Graubard B, Williamson DF, Flegal KM. Comments on “Choice of time scale and its effect on significance of predictors in longitudinal studies” by Michael J. Pencina, Martin G. Larson and Ralph B. D’Agostino. Stat Med. 2009;28(8):1315–7. doi: 10.1002/sim.3620 19288532 PMC2674613

[56] Friedman J, Hastie T, Tibshirani R. Regularization Paths for Generalized Linear Models via Coordinate Descent. J Stat Softw. 2010;33(1):1–22. doi: 10.18637/jss.v033.i01 20808728 PMC2929880

[57] Tay JK, Narasimhan B, Hastie T. Elastic net regularization paths for all generalized linear models. J Stat Softw. 2023;106:1. doi: 10.18637/jss.v106.i01 37138589 PMC10153598

[58] R Core Team. R: A language and environment for statistical computing [Internet]. 2021. Available from: https://www.R-project.org/

[59] Wickham H, François R, Henry L, Müller K, Vaughan D. dplyr: A Grammar of Data Manipulation. 2021.

[60] Wickham H, Girlich M, Ruiz E. dbplyr: A “dplyr” back end for databases. 2021.

[61] Henry L, Wickham H. rlang: Functions for Base Types and Core R and “Tidyverse” Features [Internet]. 2023. Available from: https://CRAN.R-project.org/package=rlang

[62] Dowle M, Srinivasan A. data.table: Extension of `data.frame`. 2021.

[63] Wickham H, Vaughan D, Girlich M. Tidyr: Tidy Messy Data. 2021.

[64] Wickham H, Henry L. purrr: Functional Programming Tools [Internet]. 2023. Available from: https://CRAN.R-project.org/package=purrr

[65] Mount J, Zumel N. Wrap R tools for debugging and parametric programming. 2021.

[66] Wickham H. Stringr: simple, consistent wrappers for common string operations. 2019.

[67] Russell N. hashmap: The Faster Hash Map [Internet]. 2017. Available from: https://github.com/nathan-russell/hashmap

[68] Borchers HW. pracma: Practical Numerical Math Functions. 2021.

[69] Silge J, Chow F, Kuhn M, Wickham H. rsample: General Resampling Infrastructure. 2021.

[70] Kaplan J. fastDummies: Fast Creation of Dummy (Binary) Columns and Rows from Categorical Variables. 2020.

[71] Bulow E. coder: Deterministic Categorization of Items Based on External Code Data [Internet]. 2023. Available from: https://docs.ropensci.org/coder/

[72] van Buuren S, Groothuis-Oudshoorn K. mice: Multivariate Imputation by Chained Equations in R [Internet]. J Stat Softw. 2011;45:1–67. https://www.jstatsoft.org/v45/i03/

[73] Hastie T, Tibshirani R, Narasimhan B, Chu G. Impute: Imputation for microarray data. 2021.

[74] Therneau TM. A Package for Survival Analysis in R [Internet]. 2021. Available from: https://CRAN.R-project.org/package=survival

[75] Kuhn M. caret: Classification and Regression Training [Internet]. 2021. Available from: https://github.com/topepo/caret/

[76] Yao W, Frydman H, Larocque D, Simonoff JS. LTRCforests: Ensemble Methods for Survival Data with Time-Varying Covariates. 2021. 10.1177/09622802221111549 35895510

[77] Fu W, Simonoff JS. Survival trees for left-truncated and right-censored data, with application to time-varying covariate data. Biostatistics. 2017;18(2):352–69. doi: 10.1093/biostatistics/kxw047 28025180

[78] Corporation M, Weston S. doParallel: Foreach Parallel Adaptor for the “parallel” Package. 2020.

[79] Microsoft WS. foreach: Provides Foreach Looping Construct [Internet]. 2020. Available from: https://github.com/RevolutionAnalytics/foreach

[80] Bengtsson H. A Unifying Framework for Parallel and Distributed Processing in R using Futures [Internet]. arXiv [cs.DC]. 2020. Available from: http://arxiv.org/abs/2008.00553

[81] Gaujoux R. rngtools: Utility Functions for Working with Random Number Generators [Internet]. 2020. Available from: https://renozao.github.io/rngtools

[82] Gaujoux R. doRNG: Generic Reproducible Parallel Backend for “foreach” Loops [Internet]. 2020. Available from: https://renozao.github.io/doRNG

[83] Daróczi G. logger: A Lightweight, Modern and Flexible Logging Utility [Internet]. 2021. Available from: https://daroczig.github.io/logger/

[84] Wickham H. ggplot2: Elegant Graphics for Data Analysis [Internet]. Springer-Verlag New York. 2016. Available from: https://ggplot2.tidyverse.org

