# Supplementary material for: Prognostic pan-cancer and single-cancer models: A large-scale analysis using a real-world clinico-genomic database
Source: PLoS One. 2026 Feb 13;21(2):e0341355. doi: 10.1371/journal.pone.0341355 (PMC12904442; doi:10.1371/journal.pone.0341355)
Supplement: S1 File — Table S3 is provided separately as an Excel file. (DOCX) [file pone.0341355.s001.docx]

**Supporting Information**

**for**

**Prognostic pan-cancer and single-cancer models: A large-scale analysis using a real-world clinico-genomic database**

Sarah F. McGough, Svetlana Lyalina, Devin Incerti, Yunru Huang, Stefka Tyanova, Kieran Mace, Chris Harbron, Ryan Copping, Balasubramanian Narasimhan^¶^, Robert Tibshirani^¶^

^¶^ denotes equal contribution

[**SI Tables & Figures 2**](#_heading=h.ye45ds7bcv2)

[Table S1. Summary of features included in prognostic models. 2](#_heading=h.pdeq2pxbhasj)

[Table S2. Summary of prognostic model feature sets. 4](#_heading=h.wf3cb83r6dyt)

[Table S3. List of the 354 variables selected by the pan-cancer “full” model. 5](#_heading=h.l8r2ifhe3r3)

[Provided for download as Table_S3.csv. 5](#_heading=h.c2x18wl782kt)

[Table S4. List of cancer-specific variables. Provided for download as Table_S4.xlsx. 5](#_heading=h.i2x4dadr0rfh)

[Figure S1-S5. Pan-cancer and single-cancer risk stratification plots for all cancer types. 6](#_heading=h.bjq3y3la35b6)

[Figure S6. Integrated Brier Score (IBS) for pan-cancer and single-cancer (A) Benchmark, (B) ROPRO-like, and (C) Full models. Lower IBS is indicative of better model calibration. Cancer types are arranged on the x-axis from largest to smallest sample size. 11](#_heading=h.cflxxwxp3irw)

[Figures S7-9. Top 25 clinico-genomic predictors in each single-cancer model. Predictors are ordered on the y-axis by descending coefficient (log hazard ratio). 12](#_heading=h.g23rhxcdc9a9)

[Figure S10. Number of variables selected by each single-cancer “full” model as a function of sample size. Cancer types are arranged on the x-axis from smallest to largest sample size. 15](#_heading=h.p9isi37x8xbl)

[Figure S11. Comparison of left-truncated right-censored forest (LTRCF) model performance for single-cancer and pan-cancer training cohorts with respect to the (A) c-index and (B) integrated brier score (IBS). Cancer types are arranged on the x-axis from largest to smallest sample size. 16](#_heading=h.axt5j5lsye4e)

[Figure S12. Top 10 variants associated with node2vec dimensions. Displayed node2vec dimensions are a subset of the full 128 that were chosen at least once by any model. Association was determined by the BIOT method. 17](#_heading=h.vgd4hj5os9dx)

[Figure S13. Comparison of model performance between the stratified and non-stratified Cox models with respect to (A) c-index and (B) integrated Brier score (IBS). Cancer types are arranged on the x-axis from largest to smallest sample size. 18](#_heading=h.3gytei800iij)

[**SI Materials & Methods 19**](#_heading=h.8oz8hh6vkgm)

[Adaptation of the ROPRO prognostic model 19](#_heading=h.cmf1sqyep4i)

[Investigation of alternative model fitting approaches that support interactions. 19](#_heading=h.vofg3cff1hig)

####

## SI Tables & Figures

#### **Table S1.** Summary of features included in prognostic models.

| **Modality** | **# Features (raw)** | **# Features (one-hot encoded)** | **Type** | **Description** |
| --- | --- | --- | --- | --- |
| Clinical/demographic | 17 | 32 | Categorical, Continuous | Baseline clinical/demographic variables :   - Age at index date (continuous, years) - Gender (Male/Female) - Race (White/Non-white) - BMI (Normal, underweight, overweight, obese, unknown) - ECOG (1-5) - Smoking status (History of smoking, No history of smoking) - Cancer type - Cancer stage at diagnosis (0, I, II, III, IV) - Advanced/Metastatic disease at baseline (True/False) - Insurance type (Commercial Health Plan, Self Pay, Medicare, Government Program, Patient Assistance Program) - Socioeconomic status index (SES quintile based on 2015-2019 Census, 1-5) - Time from diagnosis to genomic test (days) |
| Labs and vital signs | 412 | 412 | Continuous | Time series summaries (median, min, max, proportion abnormal, slope, variance) of laboratory tests and vital signs:  alanine_aminotransferase_alt_or_sgpt  albumin_serum  alkaline_phosphatase_alp  aspartate_aminotransferase_ast_or_sgot  bilirubin_total_serum  calcium_serum  creatinine_serum  glucose  hematocrit  hemoglobin_whole_blood  lymphocyte_count_fractionated  lymphocyte_count_absolute  monocyte_count  platelet_count  proteintotal_serum  red_blood_cell_count  urea_nitrogen  white_blood_cell_count  monocyte_fractionated  neutrophil_count_absolute  body_height  body_temperature  body_weight  diastolic_blood_pressure  heart_rate  systolic_blood_pressure |
| Treatment | 32 | 32 | Binary, Continuous | Indicators for each unique drug category received during frontline therapy and treatment at an academic center; continuous variable describing number of unique drugs received in frontline therapy, year of frontline therapy, time from diagnosis to first treatment.  Drug categories:  anesthetic  antiinfective  antianemic  antibodyconjugate  anticholinergic  antiemetic  antihyperglycemic  antineoplastic  biphosphonate  chemotherapy  cytoprotective  glucocorticoid  goutandhyperurecemiaagent  growthfactor  hematologicalagent  hormone  hyperglycemic  hypoxiainduciblefactorhifinhibitor  immunosuppressive  immunotherapy  monoclonalantibody  other  painagent  protonpumpinhibitor  radiopharmaceutical  solutionfluid  targetedbiologic  targetednonbiologic |
| Genomic | 1,511 | 1,513 | Categorical, Continuous | Binary alteration status (short variant "SV", copy number "CN", or rearrangement "RE") of genes; Raw variables are binary, while imputed ones are continuous due to the nature of KNN imputation (the resulting value reflects level of agreement between nearest neighbors). Variables consist of all combinations of HUGO gene symbol and variant type (SV, CN, RE) that were assayed by any Foundation Medicine test.  Pathway affected status (“signature”) - biological pathways are designated as affected if any of their constituent genes have any kind of alteration.  Node2Vec (“hp_n2v”) 128-dimensional embedding vector averages of all genes altered in a sample. See Figure S12.  Non-alteration related features derived from or associated with Foundation Medicine data: tissue tumor mutational burden (tTMB), tumor purity (computationally derived), PDL1 status, estimated ancestry (fractional assignment to 5 superpopulations) |
| Cancer-specific | 87 | 146 | Categorical, Continuous | Prognostic factors relevant to one or more cancer types, including: sites of metastases, extranodal sites, disease subtypes, cancer-specific lab test results, disease-specific histologies. Full list provided in Table S4. |
| **Total** | 2,059 | 2,135 |  |  |
|  |  |  |  |  |
| BMI: Body mass index; ECOG: Eastern Cooperative Oncology Group; HUGO: HUman Genome Organization. | | | | |

####

#### **Table S2.** Summary of prognostic model feature sets.

| **Model** | **# Features** | **Description** | **Feature set** |
| --- | --- | --- | --- |
| Benchmark | 9 | Collection of variables commonly collected in clinical practice | Cancer type, Age, Race, Gender, Smoking status, Baseline ECOG, Cancer stage at diagnosis, Time from diagnosis to frontline treatment, Time from diagnosis to genomic test |
| ROPRO-like | 29 | All variables included in the ROPRO model (Becker et al. 2020) with <= 30% missing in the database, plus time from diagnosis to genomic test (to adjust for delayed entry). | Cancer type, Age, Gender, Smoking status, Baseline ECOG, Cancer stage at diagnosis, BMI, Body weight, Body height, Heart rate, Hemoglobin, Systolic blood pressure, Diastolic blood pressure, Urea nitrogen, ALP, ALT, AST, Calcium, Creatinine, Total protein, Bilirubin, Albumin, Hematocrit, Glucose, Platelet count, Lymphocyte count, Monocyte count, Neutrophil count, Time from diagnosis to genomic test |
| Full | 2,059 | All variables derived from the clinico-genomic database. | All clinical and genomic predictors |

#### **Table S3.** List of the 354 variables selected by the pan-cancer “full” model.

#### Provided for download as Table_S3.csv.

#### **Table S4.** List of cancer-specific variables. Provided for download as Table_S4.xlsx.

#### **Figure S1-S5.** Pan-cancer and single-cancer risk stratification plots for all cancer types.

**Figure S1.** Pan-cancer and single-cancer risk stratification plots for Non-Small Cell Lung, Colorectal, and Breast cancers.

**
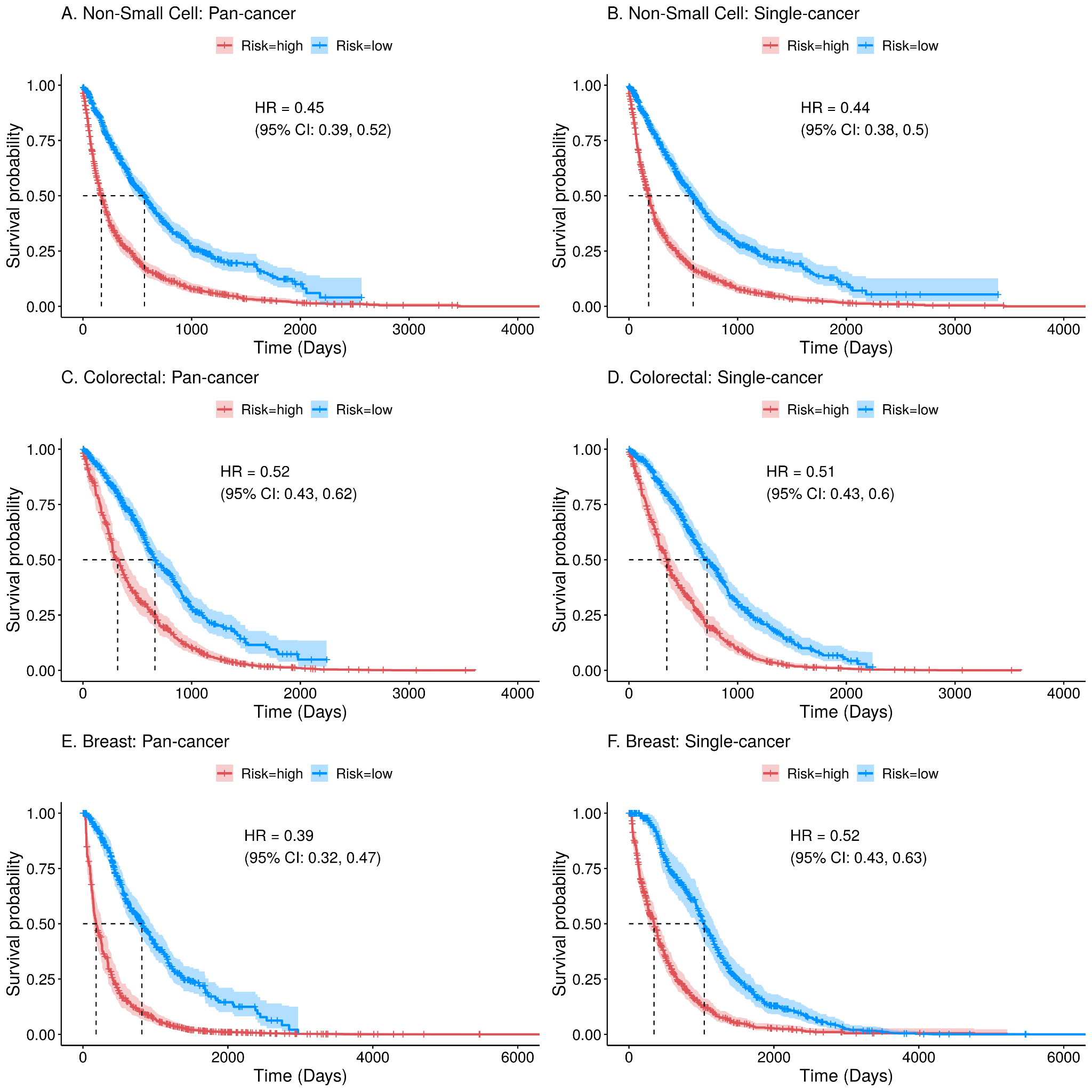
**

**Figure S2.** Pan-cancer and single-cancer risk stratification plots for Ovarian, Pancreatic, and Gastric cancers.

**
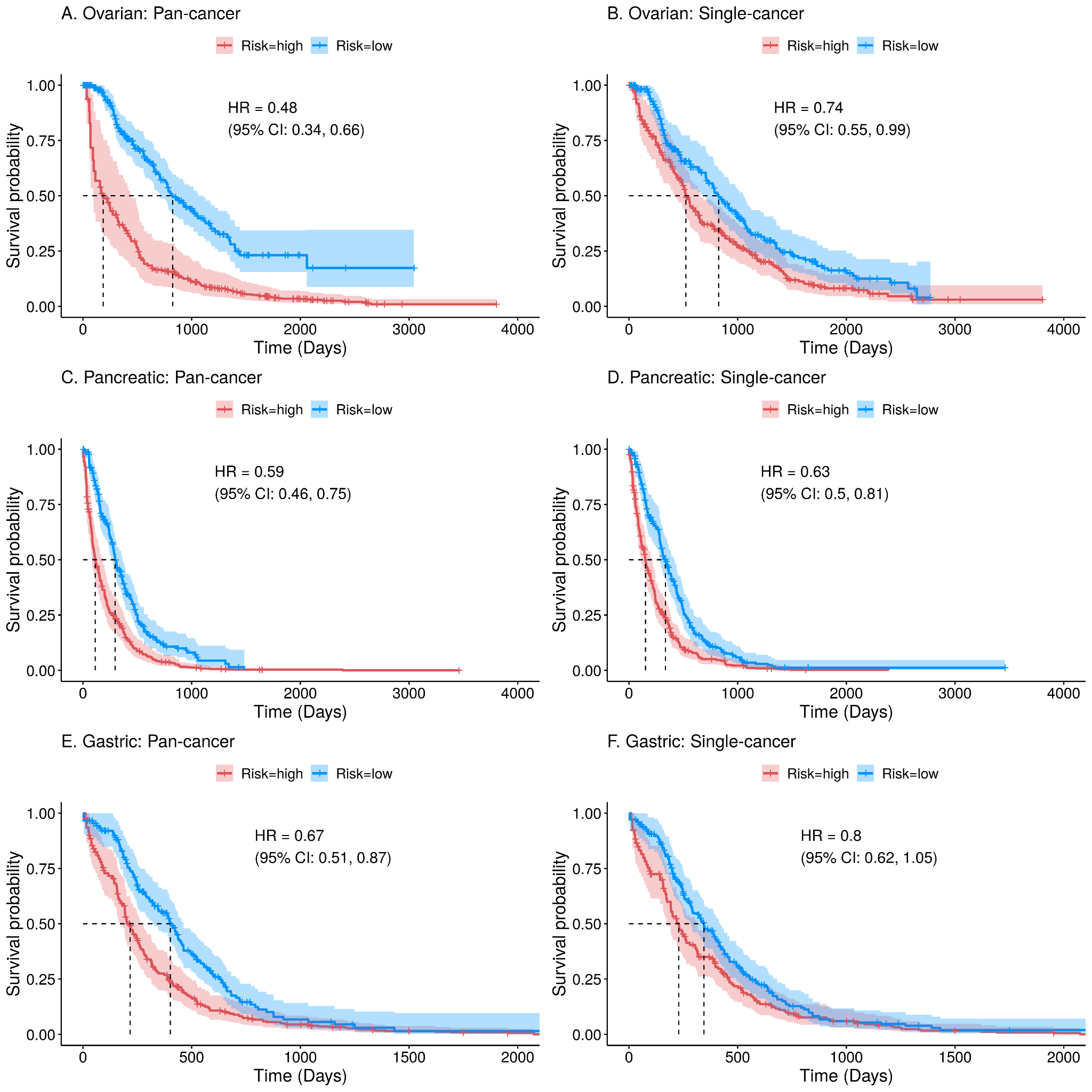
**

**Figure S3.** Pan-cancer and single-cancer risk stratification plots for Prostate, Urothelial, and Melanoma cancers.

**
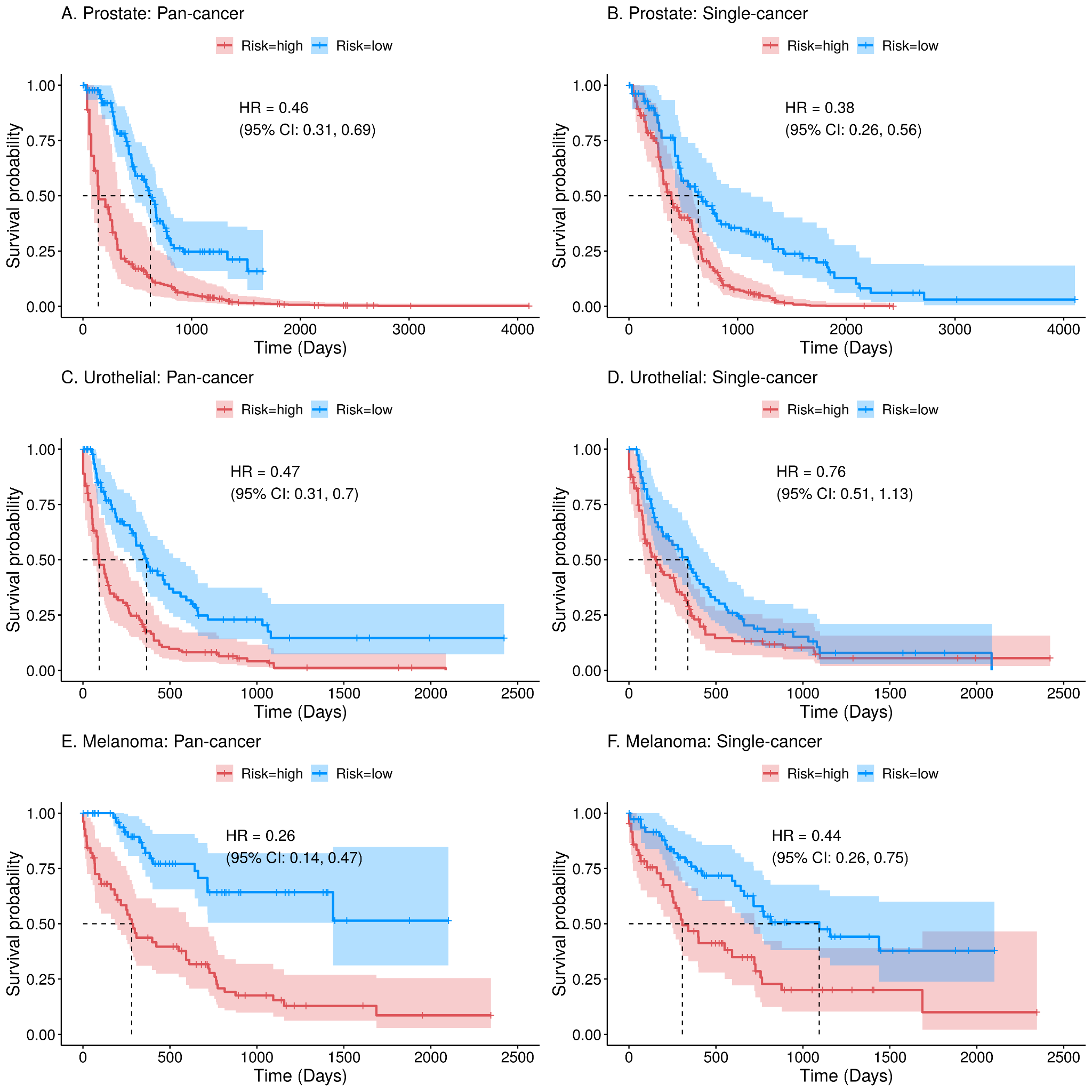
**

**Figure S4.** Pan-cancer and single-cancer risk stratification plots for Renal, Small Cell, and Head and Neck cancers.
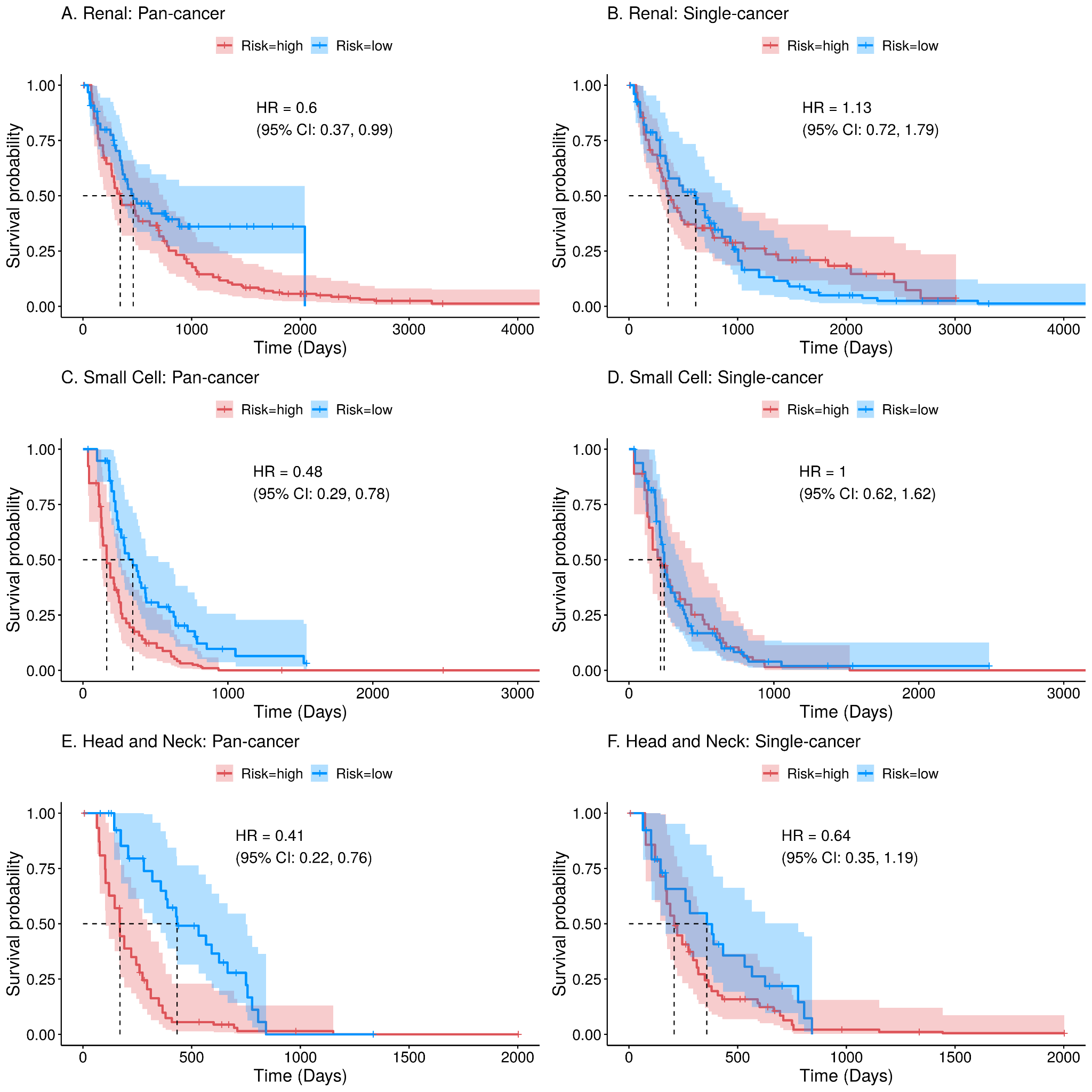


**Figure S5.** Pan-cancer and single-cancer risk stratification plots for Multiple Myeloma, Hepatocellular Carcinoma, DLBCL, and CLL.

**
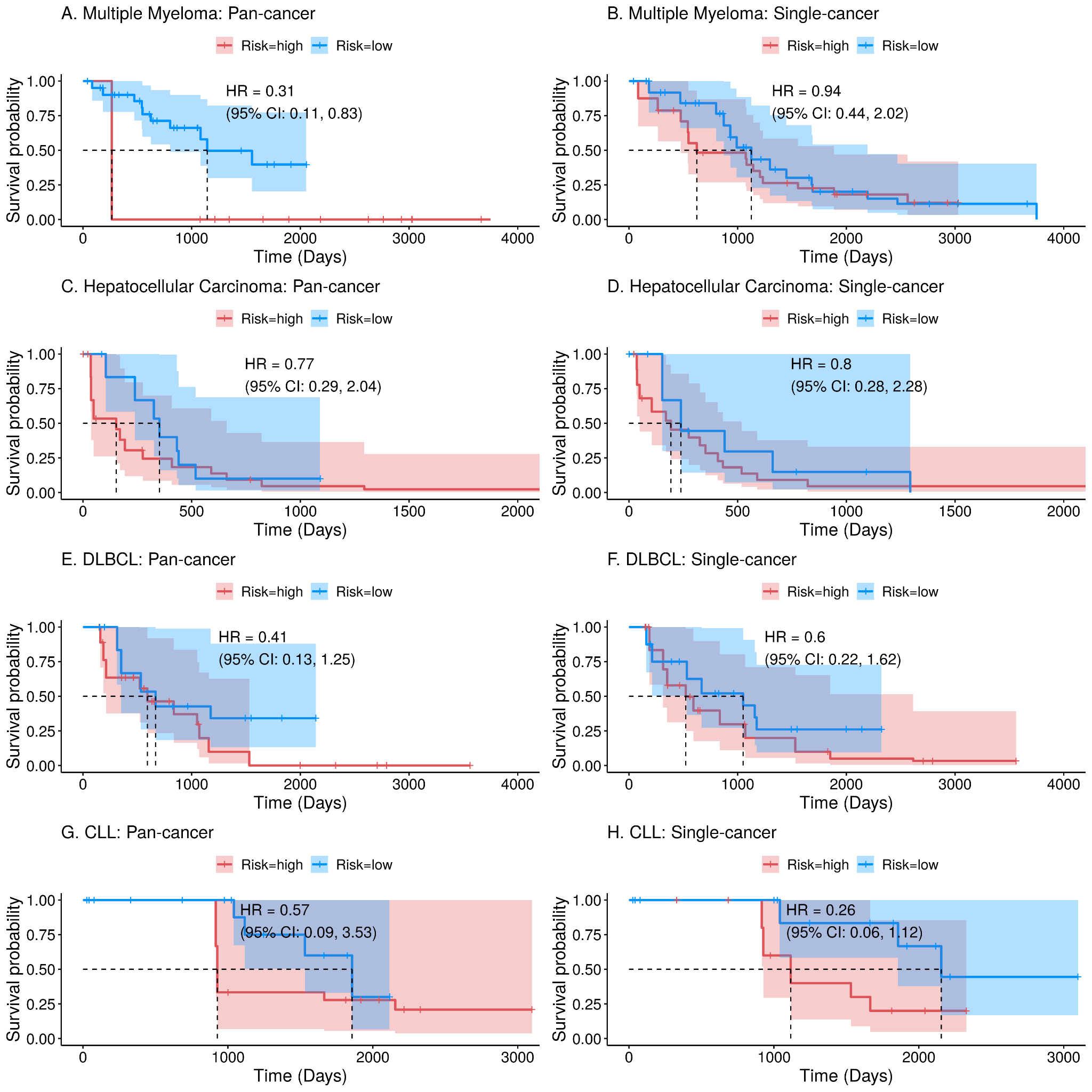
**

#### **Figure S6.** Integrated Brier Score (IBS) for pan-cancer and single-cancer (A) Benchmark, (B) ROPRO-like, and (C) Full models. Lower IBS is indicative of better model calibration. Cancer types are arranged on the x-axis from largest to smallest sample size.


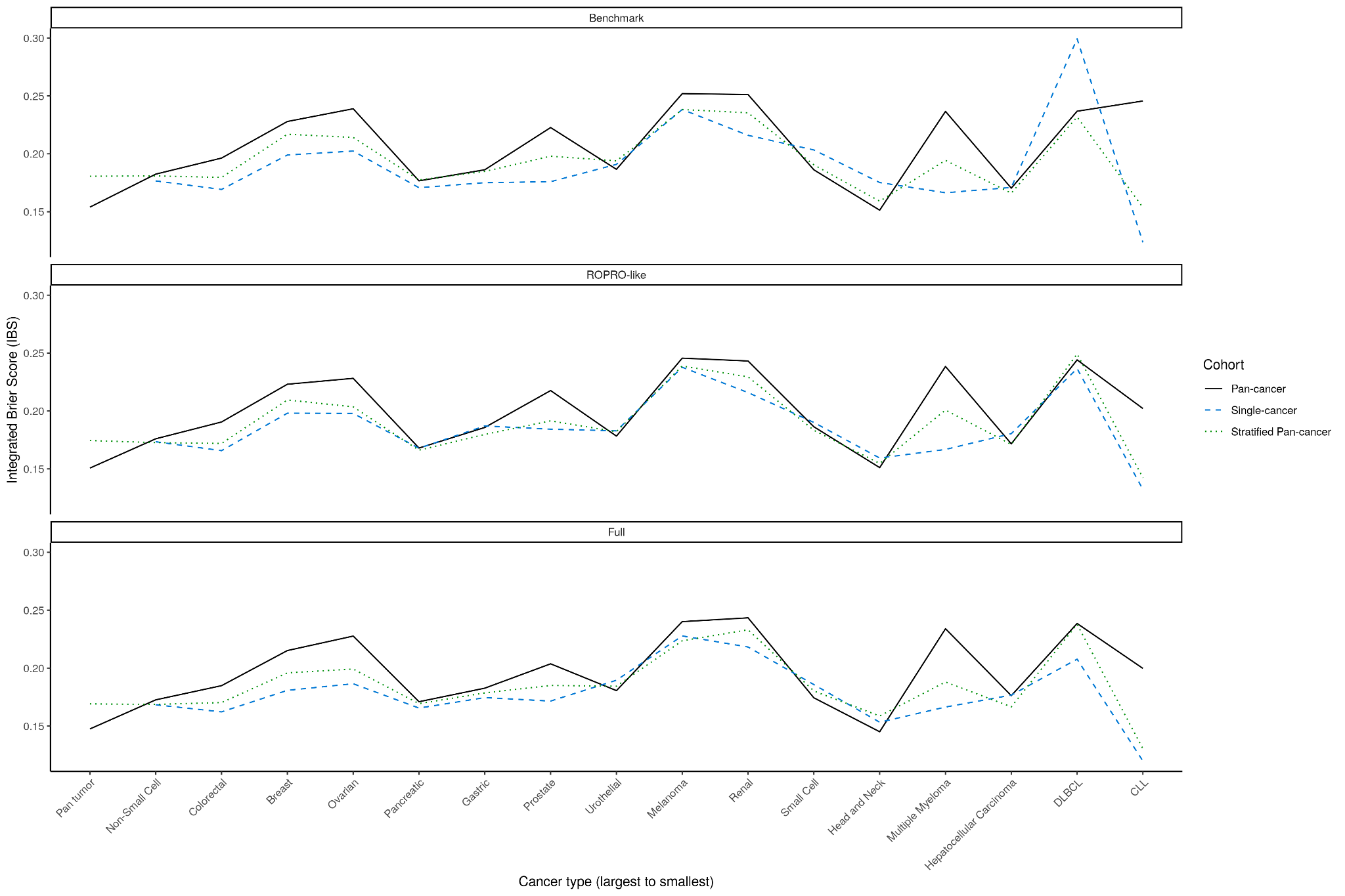


#### **Figures S7-9.** Top 25 clinico-genomic predictors in each single-cancer model. Predictors are ordered on the y-axis by descending coefficient (log hazard ratio).


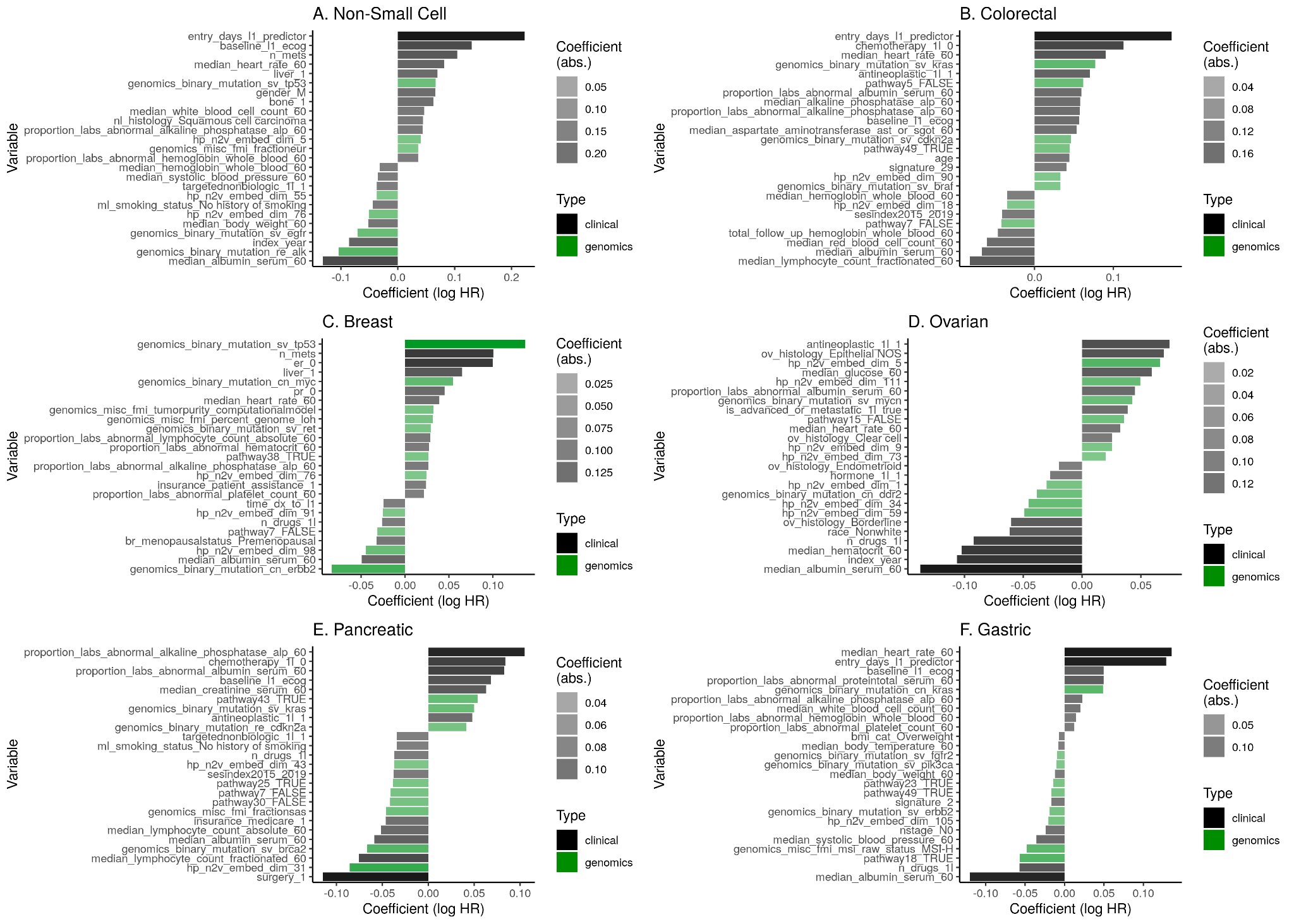


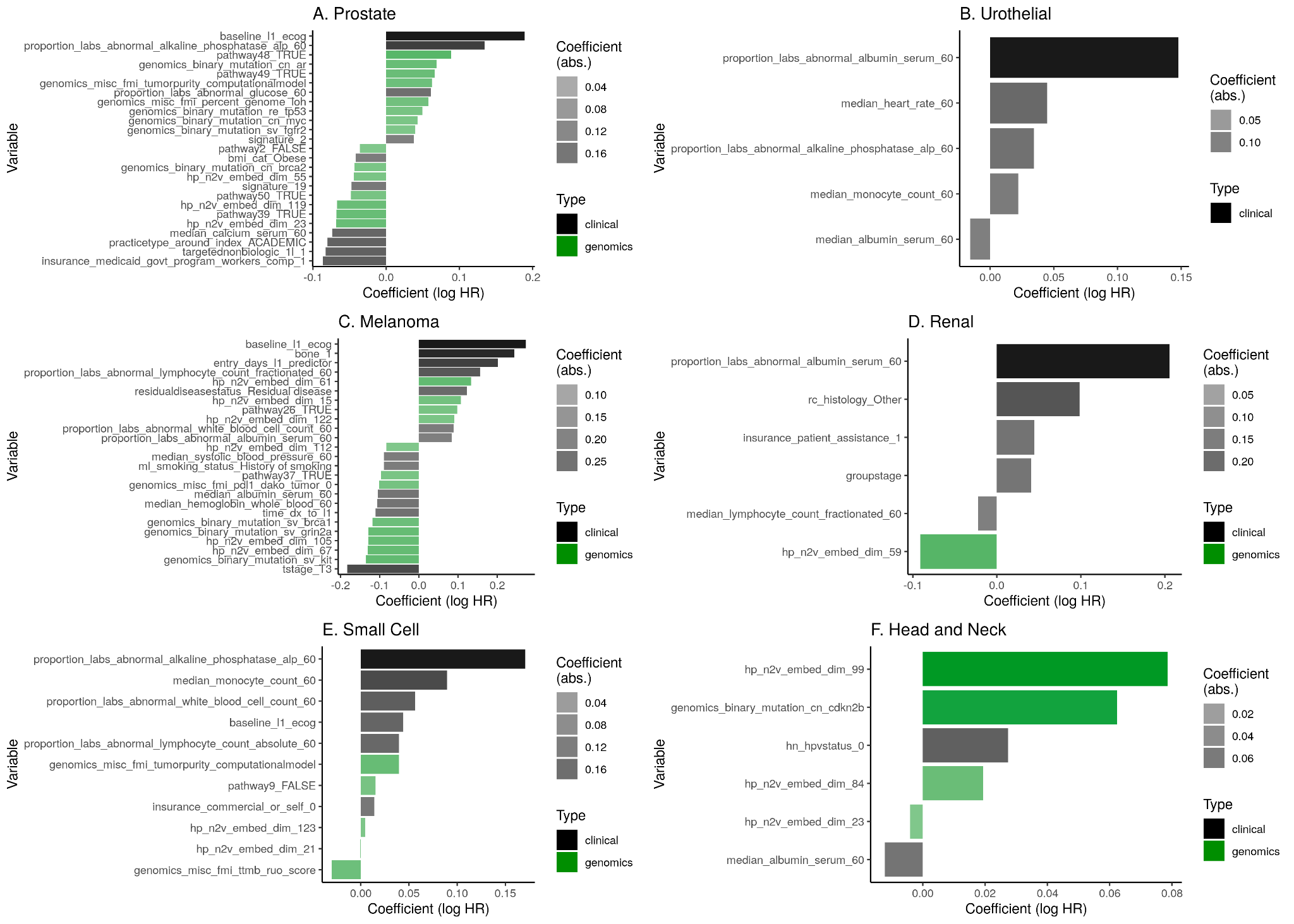


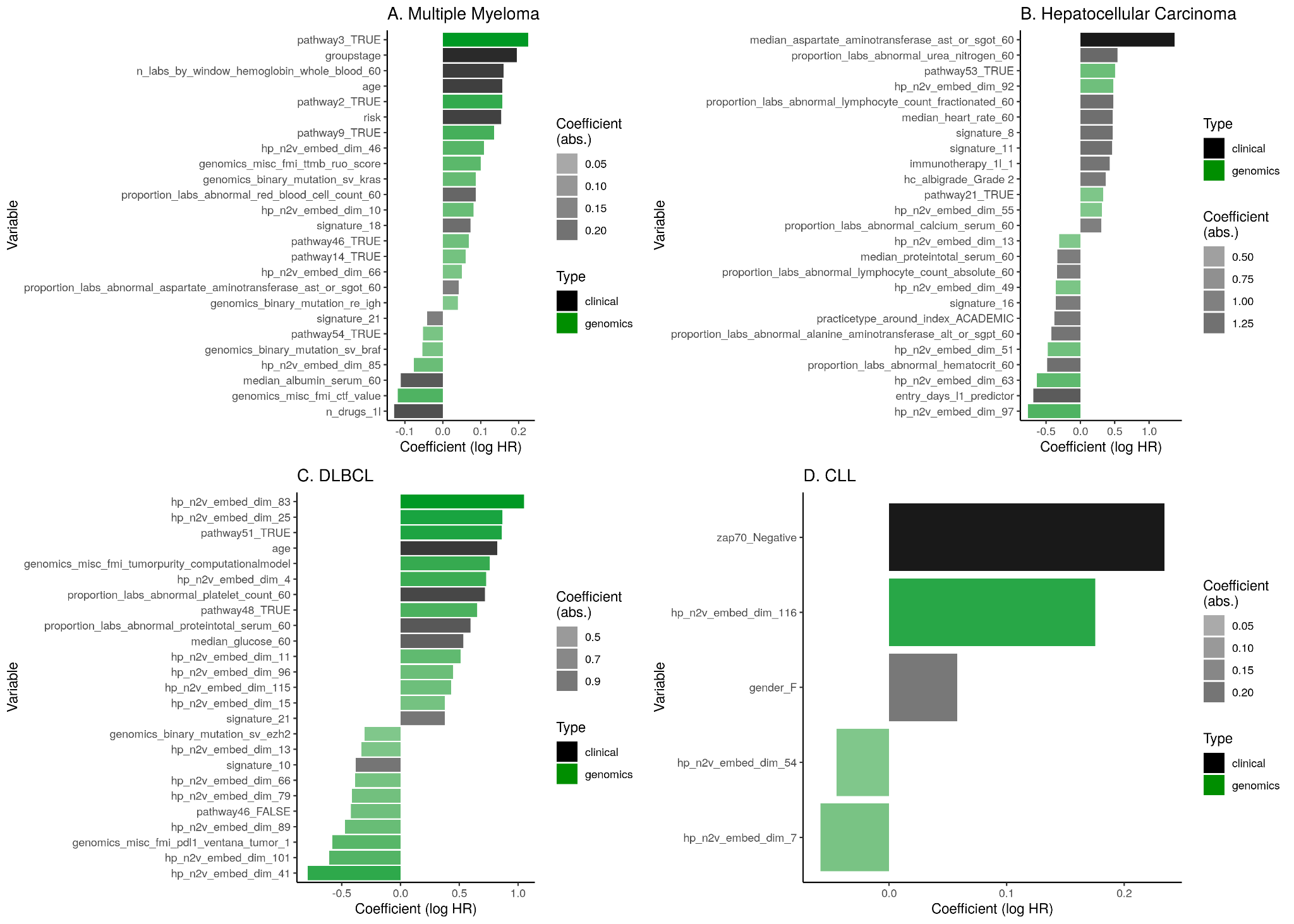


#### **Figure S10.** Number of variables selected by each single-cancer “full” model as a function of sample size. Cancer types are arranged on the x-axis from smallest to largest sample size.


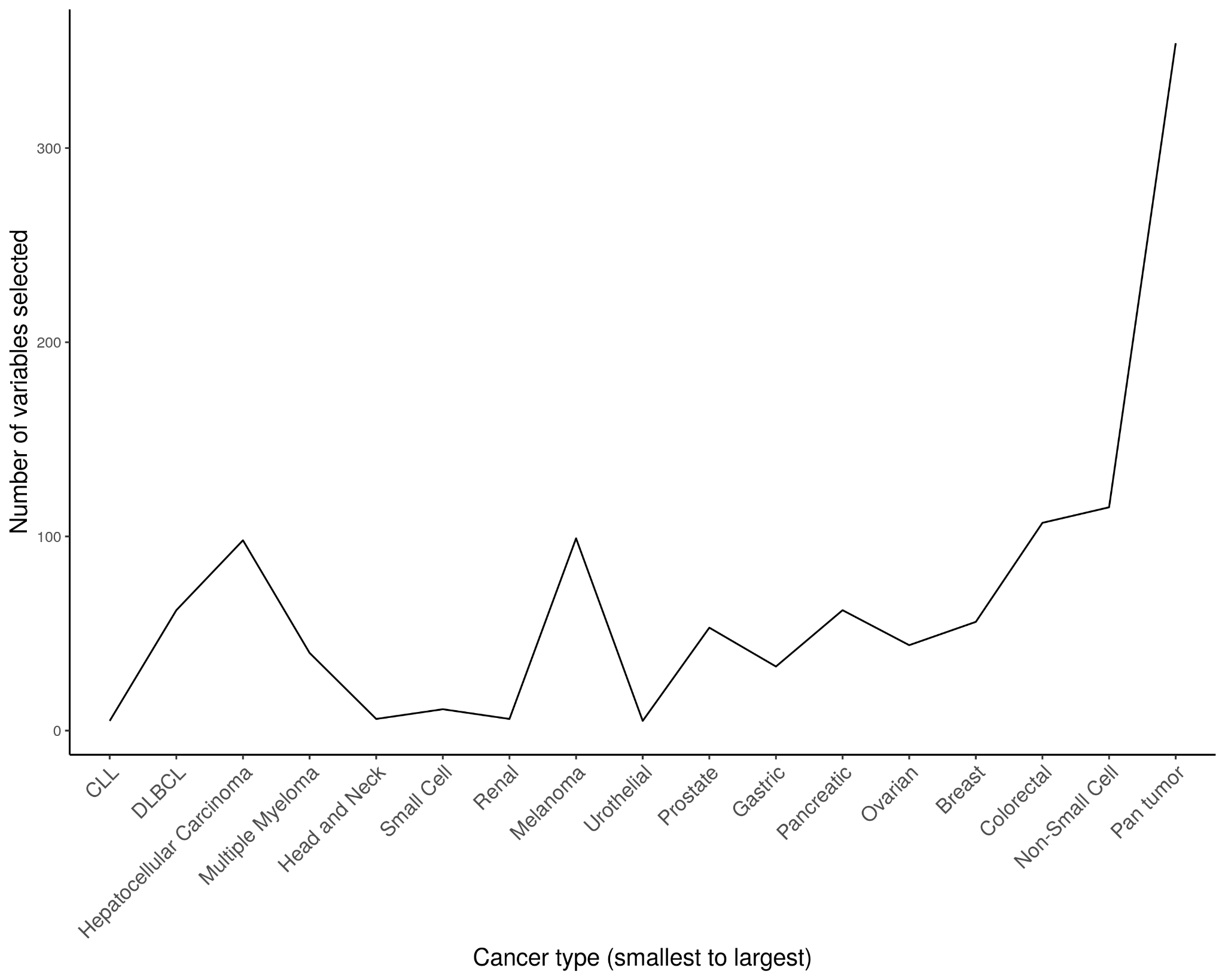


#### **Figure S11.** Comparison of left-truncated right-censored forest (LTRCF) model performance for single-cancer and pan-cancer training cohorts with respect to the (A) c-index and (B) integrated brier score (IBS). Cancer types are arranged on the x-axis from largest to smallest sample size.

**
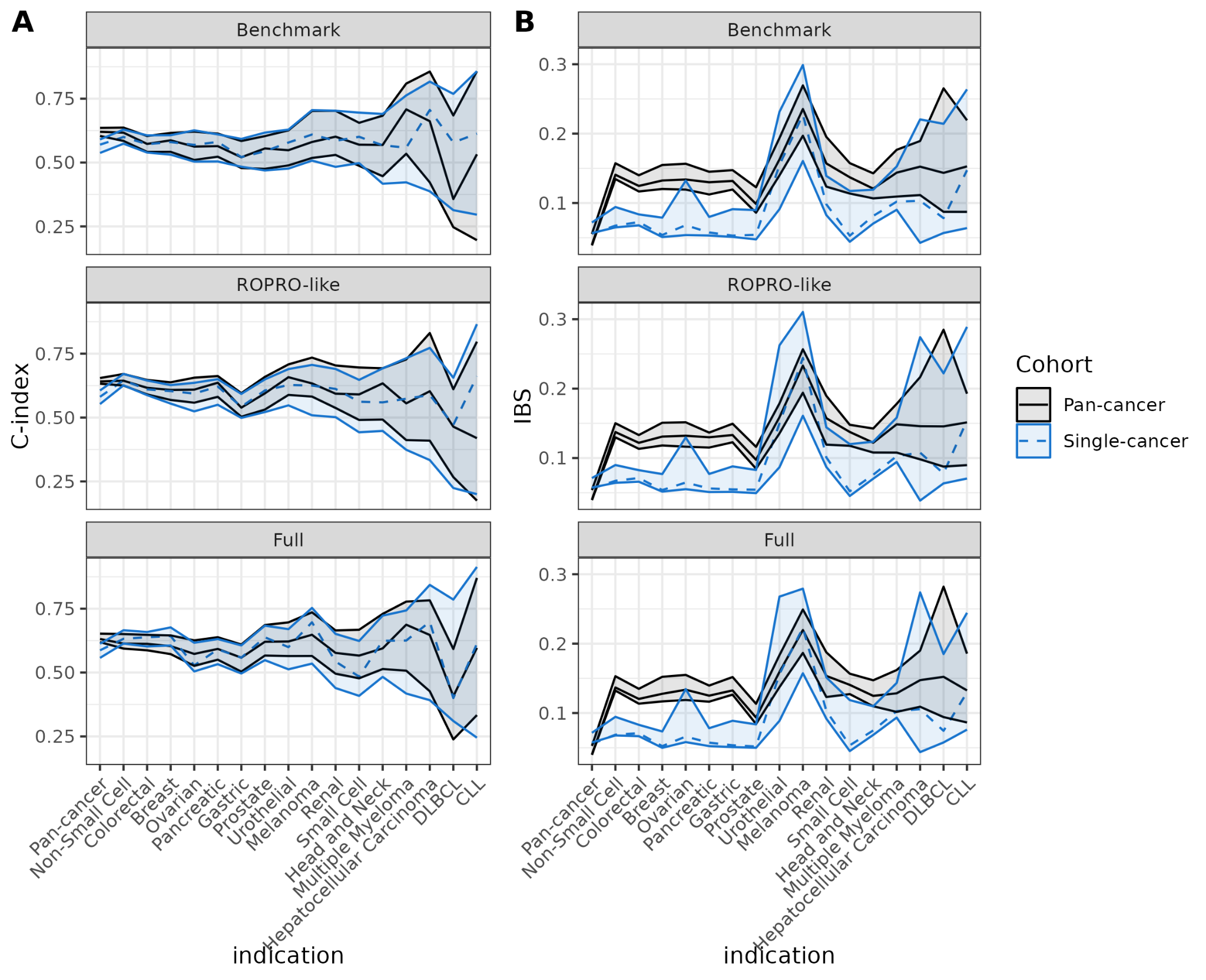
**

#### **Figure S12. Top 10 variants associated with node2vec dimensions**. Displayed node2vec dimensions are a subset of the full 128 that were chosen at least once by any model. Association was determined by the BIOT method.**
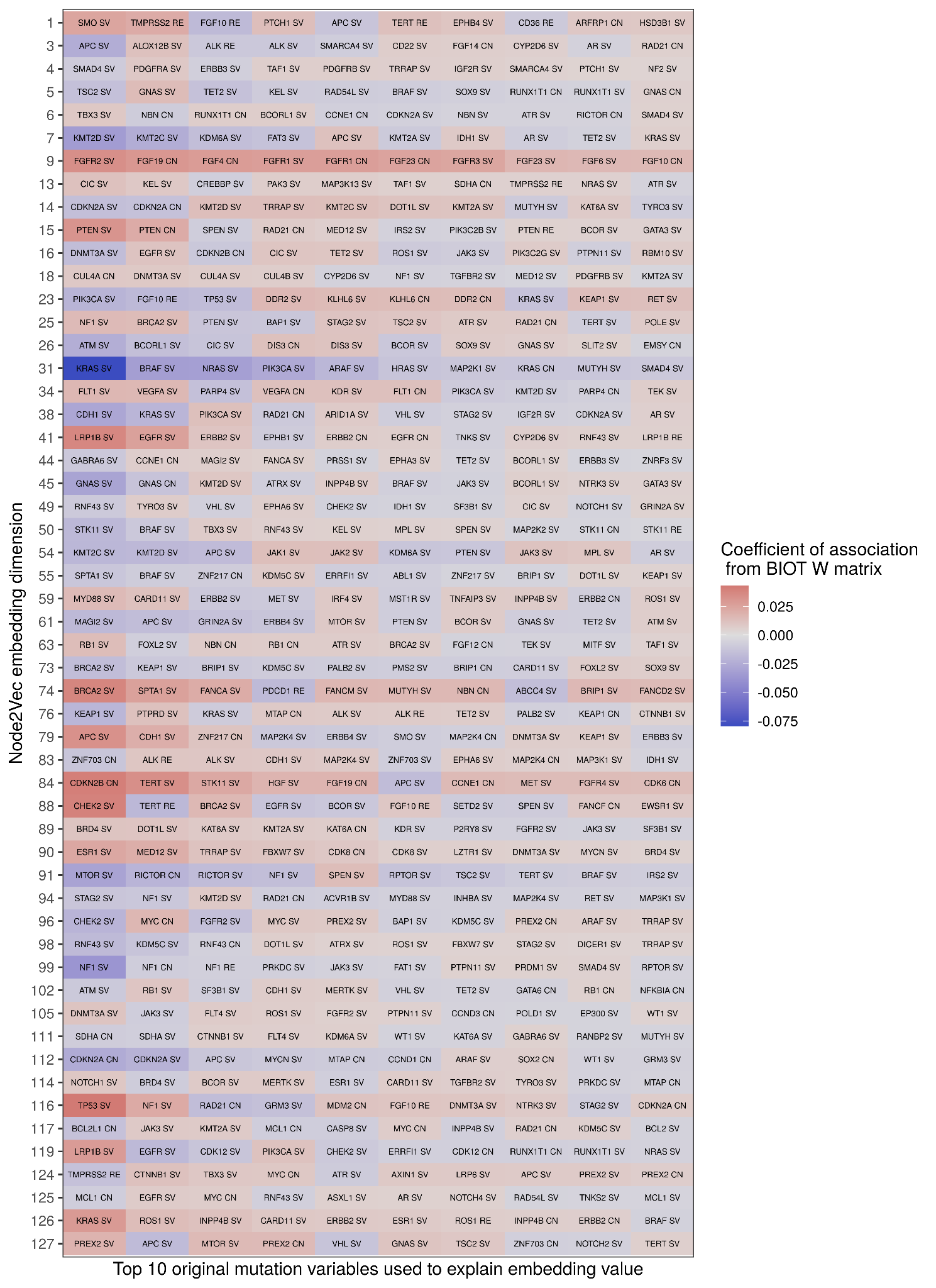
**

#### **Figure S13.** Comparison of model performance between the stratified and non-stratified Cox models with respect to (A) c-index and (B) integrated Brier score (IBS). Cancer types are arranged on the x-axis from largest to smallest sample size.

**
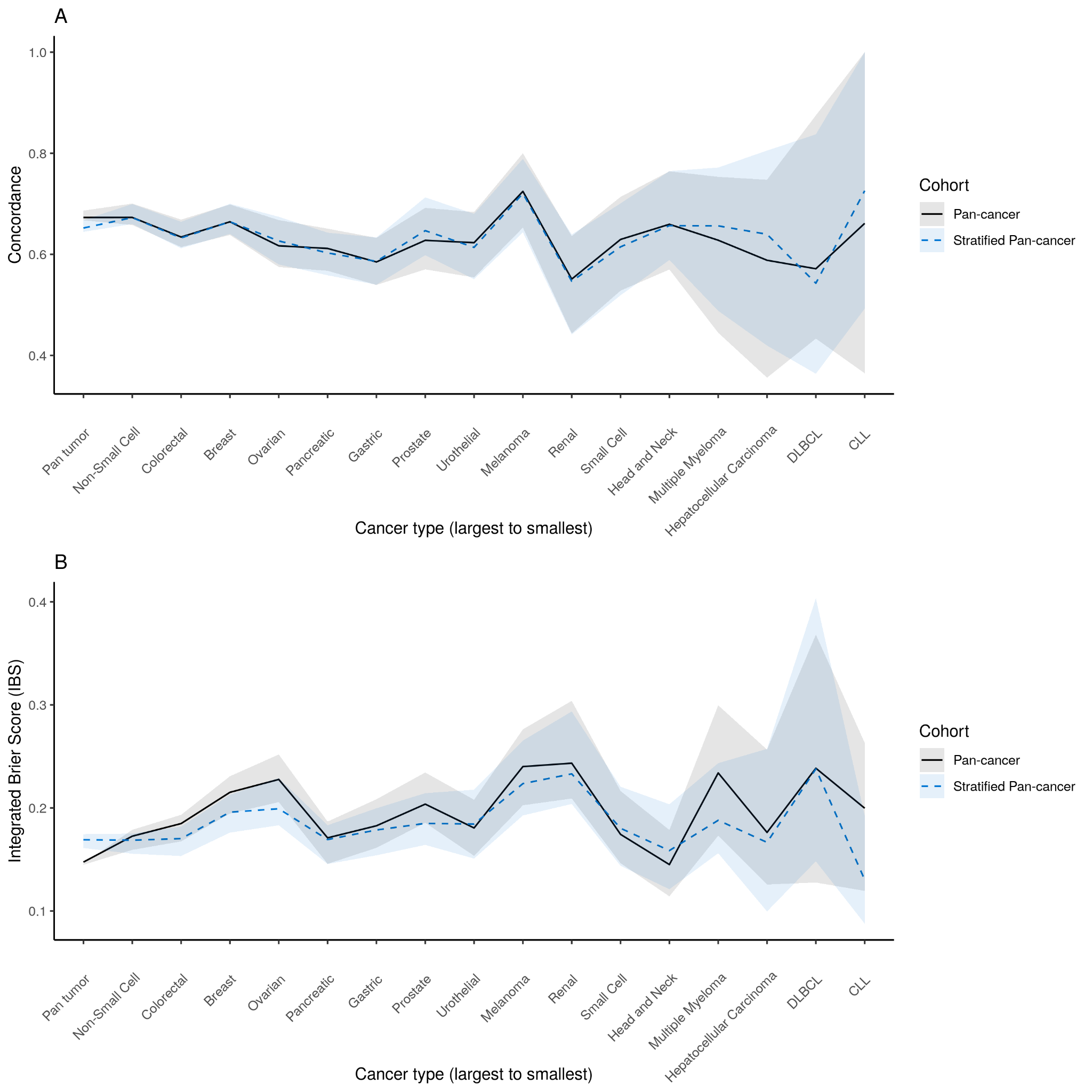
**

## SI Materials & Methods

#### ***Adaptation of the ROPRO prognostic model***

The ROPRO (Real wOrld PROgnostic score) was developed by by Becker et al^9^ and is composed of 27 clinical and demographic variables derived from the Flatiron Health electronic health record de-identified database.

We developed a “ROPRO-like” model, intended to adapt the ROPRO model using the available data in the clinico-genomic database (Table S2). However, our approach deviates in several ways:

1. To remain consistent with the rest of our benchmark models, we allowed missingness in the selected variables of up to 30% whereas the ROPRO model allowed missingness up to 75%. As a result, our “ROPRO-like” model excludes several ROPRO variables with high rates of missingness including lactate dehydrogenase (LDH), chloride, oxygen, and eosinophils.
2. Where variables like “AST-to-ALT ratio” were not available, we included the individual laboratory components, AST and ALT.
3. The ROPRO model imputed missing data using a tree-based approach, whereas we used multiple imputation to remain consistent across our benchmark models.

#### ***Investigation of alternative model fitting approaches that support interactions.***

The left-truncated right censored forests (LTRCF) R package^73,74^ was used within the same training and testing setup described for the Cox PH and Cox lasso models in the main results. The ‘mtry’ parameter was tuned via the LTRCforests::tune.ltrcrrf function (all arguments to which were set to their default values except time.eval, which was a grid from 0 to max observed time with granularity of 30 days. Default parameters: starting mtry = sqrt(number of variables), stepFactor = 2, ntreeTry = 100, bootstrap = “by.root”, samptype=”swor”, sampfrac=0.632, nsplit = 10, nodesizeTry = max of either sqrt(number of observations) or 15. Values of the linear predictor were obtained from the resulting fit using the LTRCforests:::predict.ltrcrfsrc method, and survival probabilities were calculated with the LTRCforests::predictProb method. These values were then used to calculate the final performance metrics: the concordance index and integrated Brier score.
